# Potential virus-mediated nitrogen cycling in oxygen-depleted oceanic waters

**DOI:** 10.1038/s41396-020-00825-6

**Published:** 2020-11-16

**Authors:** M. Consuelo Gazitúa, Dean R. Vik, Simon Roux, Ann C. Gregory, Benjamin Bolduc, Brittany Widner, Margaret R. Mulholland, Steven J. Hallam, Osvaldo Ulloa, Matthew B. Sullivan

**Affiliations:** 1 Department of Microbiology, The Ohio State University, Columbus, OH 43210 USA; 2 Viromica Consulting, Santiago, Chile; 3 DOE Joint Genome Institute, Berkeley, CA USA; 4 Department of Ocean, Earth and Atmospheric Sciences, Old Dominion University, Norfolk, VA USA; 5 Woods Hole Oceanographic Institution, Woods Hole, MA USA; 6 Department of Microbiology and Immunology, University of British Columbia, Vancouver, BC Canada; 7 Departamento de Oceanografía & Instituto Milenio de Oceanografía, Universidad de Concepción, Concepción, Chile; 8 Department of Civil, Environmental and Geodetic Engineering, The Ohio State University, Columbus, OH USA

**Keywords:** Virus-host interactions, Biogeochemistry, Microbial biooceanography, Microbial ecology

## Abstract

Viruses play an important role in the ecology and biogeochemistry of marine ecosystems. Beyond mortality and gene transfer, viruses can reprogram microbial metabolism during infection by expressing auxiliary metabolic genes (AMGs) involved in photosynthesis, central carbon metabolism, and nutrient cycling. While previous studies have focused on AMG diversity in the sunlit and dark ocean, less is known about the role of viruses in shaping metabolic networks along redox gradients associated with marine oxygen minimum zones (OMZs). Here, we analyzed relatively quantitative viral metagenomic datasets that profiled the oxygen gradient across Eastern Tropical South Pacific (ETSP) OMZ waters, assessing whether OMZ viruses might impact nitrogen (N) cycling via AMGs. Identified viral genomes encoded six N-cycle AMGs associated with denitrification, nitrification, assimilatory nitrate reduction, and nitrite transport. The majority of these AMGs (80%) were identified in T4-like *Myoviridae* phages, predicted to infect *Cyanobacteria* and *Proteobacteria*, or in unclassified archaeal viruses predicted to infect *Thaumarchaeota*. Four AMGs were exclusive to anoxic waters and had distributions that paralleled homologous microbial genes. Together, these findings suggest viruses modulate N-cycling processes within the ETSP OMZ and may contribute to nitrogen loss throughout the global oceans thus providing a baseline for their inclusion in the ecosystem and geochemical models.

## Introduction

Earth’s biogeochemical cycles are driven by microbial interaction networks, with significant contributions from the oceans [1, 2]. These networks and the distribution of metabolic pathways within them are modulated by environmental factors, grazing, and viral infections. Ocean viruses are abundant, kill ~20–40% of microbial cells per day, transfer genes between microbial hosts, and can more subtly impact host physiology through temperate and inefficiently lytic infections [3–9]. Virus-infected cells, termed virocells, have dramatically altered metabolic output [10–12], but can also directly impact biogeochemical cycling through virus-encoded auxiliary metabolic genes (AMGs) [9, 13–20]. Culture-based studies have shown that cyanophages (viruses of photosynthetic cyanobacteria) routinely encode core and ancillary photosynthesis genes. Virus-encoded AMGs are widespread among cyanophages with long latent periods [13], and can be expressed during infection [14–19] to enable the infecting virus to tailor host metabolism to its own needs. AMGs have been transferred to and from viruses over evolutionary timescales [13, 20], and likely provide a fitness advantage to the virus during infection [21, 22]. Metagenomic surveys using high-throughput sequencing platforms and advanced assembly methods now provide sufficient genomic coverage for taxonomic identification [23, 24], which helped identify AMGs associated with functions beyond photosynthesis, including nearly all of central carbon metabolism [25], phosphate scavenging [26, 27], sulfur cycling [28, 29], and, most recently, nitrogen (N) cycling genes including PII (a global N regulator) and *amoC* (ammonia monooxygenase subunit C) [30, 31].

Notably, however, AMG studies in the ocean have largely focused on surface ocean waters where photosynthesis is the dominant source of organic carbon. This leaves AMGs in oxygen minimum zones (OMZs), which have large impacts on climate active trace gas, nutrient cycling, and fisheries productivity [32], much less studied. Permanent OMZs make up ~8% of the total ocean volume, often have high concentrations of nitrate and nitrous oxide, and account for up to 50% of oceanic fixed-nitrogen loss—with OMZ expansion altering surface ocean primary production [32–37]. Under the suboxic conditions that characterize OMZs, nitrate serves as an alternative electron acceptor, starting the denitrification pathway that reduces nitrate to N_2_ gas [38]. In fact, most N cycling occurs in the absence of oxygen, including assimilatory and dissimilatory nitrate reduction and anaerobic ammonium oxidation (anammox) [38]. Because microorganisms that inhabit OMZs rely upon chemoautotrophy for organic carbon production and redox-coupled metabolisms that link N and sulfur cycling [32, 39, 40], we hypothesized that OMZ viruses manipulate N cycling via AMGs in ways that differ from viruses in oxic waters. This hypothesis has some support, as genes involved in ammonia assimilation, nitrate and nitrite ammonification, nitric oxide synthesis, and denitrification have been previously identified in “gene-resolved viromes” [41]. However, technological limitations at the time prevented these genes from being confirmed as viral in origin, which is critical for AMG studies given that cellular DNA is routinely encountered in viromes and can lead to rampant false discovery (see re-analyses of prior work presented in [23, 24]).

To further test the hypothesis that OMZ viruses manipulate N cycling via AMGs, we deeply-sequenced relatively quantitative viral particle metagenomes from the surface to OMZ waters of the Eastern Tropical South Pacific (ETSP) Ocean. Several N-cycle AMGs were identified, enabling us to contemplate the functional implications of viral reprogramming in relation to OMZ biogeochemistry.

## Materials and methods

### Sample collection

**Fig. 1:**
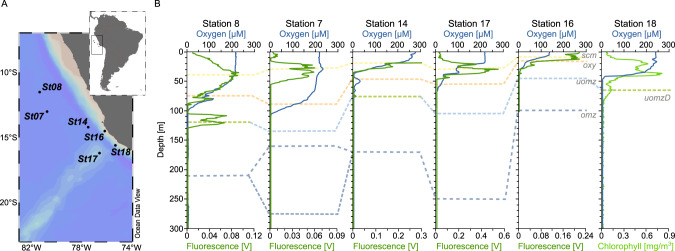
Map of the study area and vertical characterization of the sampling stations. **A** Location of stations 7, 8, 14, 16, 17, and 18, off the coast of Peru in the ETSP oxygen minimum zones (OMZ). The map was created with Ocean Data View (http://odv.awi.de). **B** Oxygen (solid blue) and fluorescence/chlorophyll (solid green, dark/light) depth profiles from each station. Fluorescence is reported instead of chlorophyll for Station 18 due to differences in the sensors used during the collection of this sample. Sampling depths are indicated with dashed lines, connected by depth category: surface chlorophyll maximum (scm) in yellow, oxycline (oxy) in orange, upper OMZ with deep chlorophyll maximum (uomzD) in green, and without DCM (uomz) in light blue, and omz core (omz) in dark blue.

In total, 22 samples were collected from six stations in the ETSP OMZ region on December 31, 2014–January 22, 2015, during the R/V Atlantis cruise AT-2626, while traversing a transect from coastal to pelagic waters. A pump-profiling system was used to collect 20 L of seawater per sample. Oxygen concentrations per sample were measured with a nanoscale sensitive, STOX oxygen sensor. Samples for NO_2_^−^, NO_3_^−^ + NO_2_^−^, and NH_4_^+^ were collected with Niskin bottles and filtered using a 0.2-µm cartridge filter. The filtrate was collected into sterile Falcon^TM^ tubes and stored upright at −20 °C until analysis. NO_2_^−^ and NO_3_^−^ + NO_2_^−^ concentrations were measured using an Astoria-Pacific autoanalyzer and standard colorimetric methods [42], and NH_4_^+^ was determined using fluorometric methods [43]. The limits of detection (LOD) for NO_2_^−^, NO_3_^−^ + NO_2_^−^, and NH_4_^+^ were 0.02 µM, 0.14 µM, and 10 nM (3σ, *n* = 7), respectively (Selden et al. submitted). Environmental features associated with each sample including oxygen, nutrient, and mineral concentrations can be found in Supplementary Table S1. Corresponding nitrate, nitrite, and ammonium concentrations were only available for half of the samples. The remaining concentrations were drawn from other samples within 10 m of the sampling depths from which our viromes were developed (denoted by an asterisk in Supplementary Table S1). Due to these sampling inconsistencies, the N-species measurements were only used qualitatively. The samples were selected corresponding to depth and habitat, including the surface chlorophyll maximum, suboxic upper oxycline, anoxic upper OMZ (with or without a deep chlorophyll maximum), and the core of the OMZ as indicated by measurements of oxygen and chlorophyll concentrations (Fig. 1 and Supplementary Table S1). A 0.2-μm filter (﻿Millipore Express Plus; Millipore) was then used for each sample to remove cells and large debris. The filtrate from each sample was retained for subsequent viral concentration and DNA sequencing.

Iron chloride flocculation was used to concentrate the viral particles from each of the 22 samples [44, 45]. Viral particles were then resuspended in an ascorbic-EDTA buffer (0.1 M EDTA, 0.2 M MgCl, 0.2 M ascorbic acid, pH 6.0). Free DNA was then removed from the viral concentrates using DNaseI at 100 U ml^−1^ concentration [25]. A wizard DNA purification kit with 1 ml of resin to 0.5 ml of sample was then used to extract the concentrated viral DNA (Promega). A CsCl density gradient was used to further purify the viral DNA in samples with >1 μg DNA (surface chlorophyll maximum samples from stations 7, 8, 14, and 16, oxycline samples from stations 14 and 16, and the core OMZ sample from station 16). Contigs derived from the CsCl-purified samples were only retained if they were the longest representative sequence of a population cluster [25]. The 22 DNase and CsCl-purified (where appropriate) samples were then used for downstream ecological analyses. Library preparation and sequencing were conducted at JGI using a Nextera kit and protocol, and an Illumina Hiseq 2000 platform.

### Assembly and processing

Data processing and metagenomic analyses were performed using high-memory computer nodes from the Ohio State Supercomputer Center [46]. Reads were split into paired and unpaired groups, adapter sequences were removed and low-quality sequencing regions below a Phred score threshold of 15, using a sliding window of four bases, were removed using a Trimmomatic version 0.33 [47], resulting in a mean read length of 149 bp. SPAdes version 3.11.1, using the –meta –sc and –careful option, was then used to assemble both paired and unpaired reads, from both samples purified with CsCl and those without, with k-mers of 21, 33, and 55 bases [48]. Population scale ecological groups were derived from these scaffolds using an in-house wrapper script for MUMmer with the nucmer package by clustering sequences at 95% ANI over 80% of the shorter sequence [49, 50]. Scaffolds that contained the same potential AMG sequence and shared an overlapping region >1 kb and 99% ANI were merged, and N-gaps in or nearby potential AMG genes were filled based on alignments against members of the same cluster. The edited scaffolds were mapped against the reads from their corresponding sample and visualized with Integrative Genomics Viewer (IGV) [51] to address even distribution of the paired reads. The edited scaffolds were identified with the letter E at the end of their name.

### Viral identification and classification

Viruses were identified among the populations larger than 5 kbp or circular and larger than 1.5 kbp using the viral identification tools VirSorter and Virfinder [52, 53]. Populations with clearly identifiable viral or viral hallmark genes in VirSorter categories 1 and 2, and populations with a Virfinder score higher than 0.9 and a *P* value < 0.05 were considered to be viral. A final size threshold of 10 kbp was implemented. Taxonomic assignment of the AMG encoding contigs was established using vConTACT v2.0 with default settings on CyVerse [54] (http://www.cyverse.org).

### Viral relative abundance and distribution

The final selection of high-confidence viral populations larger than 10 kbp was concatenated into a single reference database, used with a custom wrapper script for bowtie2 to recruit quality trimmed reads from each sample, except for the CsCl treatment samples which were replaced with their corresponding non-CsCl counterparts to avoid treatment-specific effects [55]. An in-depth analysis of the viral community ecology from the same samples revealed no detectable correlation between the minimal variation of sequencing depth and the diversity in these samples [56]. The relative abundance of each population per sample was then derived from the resulting bam files and converted into a relative abundance table using a custom wrapper script for BamM (https://github.com/ecogenomics/BamM). Coverage values as relative abundance proxies were calculated using the “tpmean” algorithm, normalized for the size of each metagenome in bases, and the length of each contig. Relative coverages were only reported for populations with read mapping coverage greater than 80% of contig length, and having at least 5× the coverage.

### AMG identification and annotation

Predicted genes were functionally annotated as done in Daly et al. [57]. Briefly, ORFs were predicted with prodigal v2.6.3 using the meta option [58]. Each predicted ORF was then screened against the KEGG, Uniref90, and InterPro database using USEARCH and Interproscan respectively [59–63]. The quality of annotation was then prescribed by a hierarchical ranking score from highest confidence to lowest confidence on a scale of A-E as follows. Annotations with a reciprocal blast hit (RBH) bitscore >350 to the KEGG database were given a score of A, RBH to the Uniref90 database with a bitscore >350 were given a score of B, the one-way blast hits to the Uniref90 database were given rank C, annotations with hits only to InterPro database were given a score of D, and those annotations with a bitscore of <60 to any database were given a rank of E [57]. Genes involved with nitrogen metabolism were then identified by manual curation of the functional annotations.

The nitrogen metabolism genes identified on viral populations were then submitted to a series of careful in silico validation steps in order to ensure that they were encoded on a bonafide viral sequence and that the functional annotations were correct. Only viral contigs larger than 10 kbp and encoding viral-like genes were reported. Where appropriate, BLAST-based homology searches and syntenic comparison with either viral or microbial references were conducted to identify the most related microbe and/or virus encoding similar metabolic and flanking genes. Genome maps and the systemic organization of related sequences were visualized using genbank files derived from NCBI for reference microbial and viral genomes, and PHANOTATE [64] for viral contigs, and the Easyfig package with tBLASTx (min. length 30 bp, max. *e*-value 0.001) [65].

Conserved residues and active sites for each predicted nitrogen metabolism-related AMG were identified using PROSITE (release 2019_02 of 13-Feb-2019, default settings, https://prosite.expasy.org/) [66] and HHpred v2.0.13 (against PDB, default settings) [67]. Binding sites were checked manually when available, and promoter/terminator regions were predicted as done by Sullivan et al. [26], with some modifications. Briefly, ORF predictions were made using PHANOTATE [64], and manually refined where alternate start sites were present, to maximize ORF size and coverage against reference genes. Bacterial sigma-70 promoters and terminators were predicted using BPROM (LDF > 2.75, Softberry, Mount Kisco, NY) [68] and TransTermHP (confidence score >90%) [69], respectively, using default parameters. Known cyanobacterial NtcA promoters were identified using the probabilistic model of NtcA-binding sites [70] that was more specifically adapted for use with marine cyanobacteria (5′-GTA-N8-TAC-3′; [71]). In addition to probability scoring cut-offs, all promoters or terminators also were required to be intergenic or within 10 bp of the start/stop of an ORF.

To avoid reporting erroneous functional annotations, based solely on sequence similarity searches, and to further support the possibility that the AMGs may be functional in the environment, we also predict the protein structure for each AMG. Secondary and tertiary structural homology searches were conducted for each AMG by first predicting the structure of the protein of interest with Phyre2 (version 2.0) [72] in expert batch submission mode. Predicted secondary structures with a 100% confidence score and alignment coverage above 70% were considered for further analyses. The most current version of SWISS-MODEL was then used to predict the quaternary structure of each protein with a Global Model Quality Estimation (GMQE) score above 0.5 [73]. Transmembrane domains were predicted with TMHMM [74].

Synonymous and non-synonymous mutations were calculated in order to determine the mode of selection acting on each protein. pN/pS values were calculated using the method from Schloissnig et al. [75], without reading coverage downsampling, and values of <0.3 were interpreted to indicate strong purifying selection.

### Phylogenetic tree generation

A phylogenetic analysis was used to further investigate the evolutionary origin of the AMGs. Sequences from this dataset were compared with the NCBI nr database [76] (blastp, the cutoff of 50 on bitscore, and 0.001 on *e*-value) to recruit closely related sequences and to add nonviral context to the phylogenetic trees. The best blast hits of each AMG, together with reference microbial sequences and previously described viral sequences (for *amoC* and *glnK*), were included in the final dataset for phylogenetic analysis. The multiple alignments and trees were built using ete3 toolkit v3.1.1 [77] with the eggnog41 pipeline, i.e., multiple alignments computed with the built-in metaligner function, automatic alignment trimming with trimAL [78], automatic model selection with ProtTest [79], and tree built with Phyml [80] with Chi^2^-based parametric branch supports. Trees were visualized using the ITOL (v3) online server [81].

## Results and discussion

### The ETSP virome dataset and overview of discovered N-related AMGs

A total of 29 viral metagenomes were sequenced from 22 samples that spanned oxic to anoxic waters across six depth profiles in the ETSP (Fig. 1A, B). The resultant 210 Gb dataset averaged ~49 M reads per virome and yielded 61,700 non-redundant, >5 kb scaffolds. Of these, 46,127 (75%) were identified as viral and 3,589 (6%) as microbial, while the remaining 11,984 scaffolds (19%) could not be identified confidently [56]. Collectively these pooled viruses recruit between 19 and 53% of the total reads, and the microbial contigs between 2 and 19%, with the rest representing unidentified contigs, or contigs smaller than 5 kb (Supplementary Fig. S1).

These high-confidence viral genomic scaffolds were screened for AMGs involved in N cycling and regulation. This screen was conducted using: (I) homology-based comparisons using USEARCH and interproscan with reference databases (KEGG, Uniprot90, Interpro), (II) analyses for conserved residues using PROSITE and HHpred, and (III) structural modeling using PHYRE2 for tertiary structures and SWISS-MODEL for quaternary structures when applicable (details in “Materials and methods”). Together these analyses revealed six AMGs— *focA, nirA, nirK, norB, amoC*, and *glnK*—, of which only two, the nitrogen regulator PII gene encoded by *glnK* and the ammonia monooxygenase encoded by *amoC*, were previously known as AMGs. Below we describe the genomic contexts and taxonomic origins of these AMGs, assess their evolutionary histories and potential ecological roles and functionality, and map their distributions within the ETSP OMZ.

#### Ferredoxin-nitrite reductase and a nitrite transporter

**Fig. 2:**
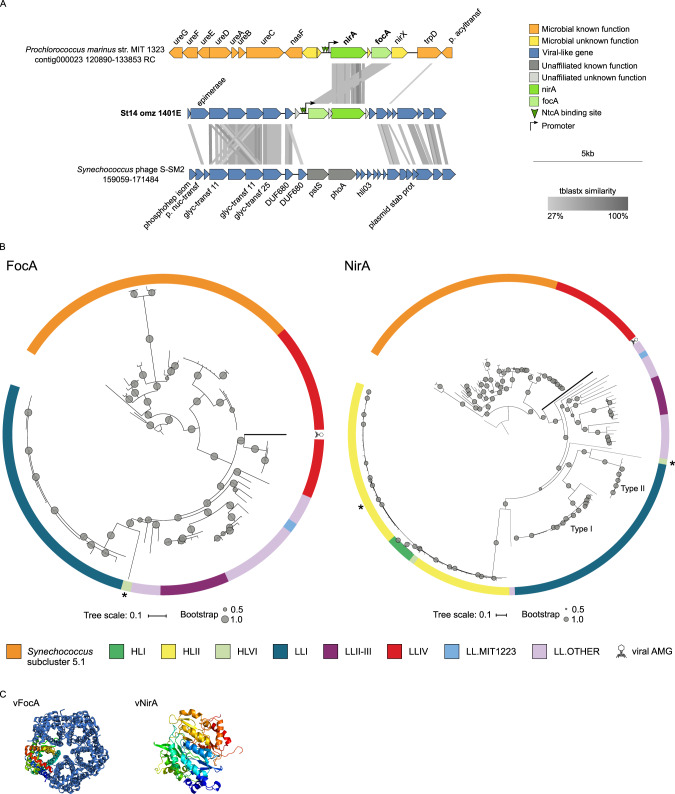
Genomic context, diversity, and protein structure of viral *focA* and *nirA*. **A** Genetic map of the scaffold encoding *nirA* and *focA* and its alignment to a reference cyanobacterial genome and a reference cyanophage genome. Detailed annotation of the ETSP viral contig can be found in Supplementary Table S2. **B** Maximum-likelihood trees from amino-acid alignments of the viral FocA or NirA found in ETSP and cyanobacterial sequences. Branches from viral AMGs found in this study are highlighted with thick lines. Internal nodes and SH-like supports are represented by proportional circles (all nodes with support <0.50 were collapsed). Asterisks indicate *Prochlorococcus* sequences where horizontal gene transfer of *nirA* and *focA* (AG-363-P06 single cell (HLVI clade)) and *nirA* (*Prochlorococcus* MIT0604 (HLII clade)) have been proposed (from refs. [87, 88]). Colors represent *Synechococcus* subcluster 5.1, and *Prochlorococcus* high-light (HL) and low-light (LL) adapted clades. **C** Quaternary structure of viral FocA and tertiary structure of viral NirA.

The first and second of the six N-cycle AMGs included a nitrite transporter gene (*focA*) and a ferredoxin-nitrite reductase gene (*nirA*), neither of which had been previously detected in viral genomes. Both genes were co-localized on the viral scaffold St14_omz_1401E (11,826 bp) that contained 20 genes, 15 of which were viral-like according to VirSorter [52], and other features ordered as follows: two predicted NtcA-binding sites, a promoter, *focA*, an unknown gene and *nirA*, though with no terminator predicted (Fig. 2A). Three lines of evidence suggest this viral scaffold originated from a T4-like cyanomyophage. First, glycosyltransferases and hypothetical protein genes surrounding *focA* and *nirA* were most closely related to those from the T4-like cyanomyophage *Synechococcus* phage S-SM2, and 5 of these genes were enriched in *Synechococcus* and/or *Prochlorococcus* T4-like phages [26] (Fig. 2A and Supplementary Table S2). Moreover, nine viral scaffolds with the most similar abundance profile as the *focA-nirA-*encoding scaffold, had a gene composition, including viral hallmark and structural genes, also associated to cyanophages, and synteny with *Synechococcus* phage S-SM2, suggesting that they all represent genome fragments of the same cyanophage population (Supplementary Fig. S2). Second, high-confidence gene-sharing network clustering (*sensu* vConTACT 2.0. [54]) placed this viral scaffold with the T4-like cyanophages (Supplementary Table S3). Consistent with this, the viral FocA and NirA were most similar to homologs in *Prochlorococcus* and *Synechococcus* (Fig. 2B, Supplementary Figs. S3 and S4), with identities of 86 and 67%, respectively, suggesting the AMGs were derived from and may function during infection in these hosts, as was observed for cyanophage photosynthesis AMGs (e.g., *hliP/psbA*/*psbD* [13, 14, 16]). Third, the putative regulation of these genes by NtcA is a common feature in cyanobacteria, where it activates genes involved in nitrogen transport and assimilation, including the *nirA* operon, heterocyst differentiation and acclimation to nitrogen starvation [82–86] (Fig. 2A).

Phylogenetic and comparative genomic analyses of these genes suggested a complicated evolutionary history. *focA* likely serves niche-defining functions in *Prochlorococcus* and *Synechococcus* as it was absent from most surface water metagenomes where nitrite is not abundant [87], and mostly absent in *Prochlorococcus* sps. from high-light (HL) clades [88]. Among cyanobacteria, regions encoding nitrate assimilation genes do not have conserved composition or order (synteny) [86]. However, the presence of these genes in multiple closely related cyanobacteria indicates that they can be horizontally transferred, perhaps even with viral versions being transferred back into the cyanobacterial genome, as observed for *psbA* [13]. These genes’ mobility is also supported by the loss and subsequent gain of *nirA* in *Prochlorococcus* HLII strains (marked in yellow in Fig. 2B and Supplementary Fig. S4), where phages may have mediated these changes given the proximity of integrase genes to *nirA* in the genome of *Prochlorococcus* MIT0604 [87]. Furthermore, evidence for *focA* and *nirA* mobility has also been suggested for the high-light adapted AG-363-P06 single cell from the HLVI clade, which has recently acquired the nitrite assimilation cassette from a low-light adapted *Prochlorococcus* [88] (Fig. 2B). Only two (out of eleven) cyanobacterial nitrate assimilation gene arrangements encoded both *nirA* and *focA*, disposed in the opposite order from the OMZ viral genomic scaffold (Fig. 2A). While the variable synteny prevents the robust reconstruction of the HGT events that led to these genes being encoded by phages, it is possible that the different placement of these AMGs in single-gene trees reflects an independent acquisition of each gene, likely from *Prochlorococcus* sps. from LLIV clade (Fig. 2B).

Evidence of function for these viral AMGs stems from in silico observations of the proteins. For FocA, known features (six transmembrane domains) and a conserved and highly charged C-terminal regulatory region [89, 90] were identified (Supplementary Fig. S5A). The viral protein was similar (86% amino-acid identity) to that in *Prochlorococcus marinus* MIT1323 and *Prochlorococcus* sp. MIT0701 (LLIV) (Fig. 2B), and the structural model was predicted with 100% confidence to be a formate/nitrite transporter (Fig. 2C and Supplementary Table S4). For NirA, known features that were identified included two nitrite/sulfite reductase ferredoxin-like half domains and a nitrite/sulfite reductase 4Fe–4S-binding site [84] (Supplementary Fig. S5B). The viral protein was similar (67% amino-acid identity) to a functionally active *Synechococcus* NirA (Fig. 2B), and the structural model was again predicted with 100% confidence to be a NirA (Fig. 2C and Supplementary Table S4). In addition, the calculated ratio of non-synonymous to synonymous polymorphisms (pN/pS) for *focA* was 0, with seven single-nucleotide polymorphisms (SNPs) identified, and 0.25 for *nirA*, with 27 identified SNPs, indicating strong purifying selection (pN/pS ratios <0.3; Supplementary Table S5) as would be expected for a gene encoding a functional protein.

Functionally, it is plausible that the acquisition of *nirA* and *focA* genes could benefit OMZ viruses. NirA is involved in assimilatory nitrite reduction (nitrite reduction to ammonia; [91]), whereas FocA is a nitrite transporter from the formate/nitrite family [92]. NirA was initially described in cyanobacteria but is widely distributed among eukaryotic algae and vascular plants [91], as well as other bacteria and archaea, as observed in the NCBI database, while FocA is found in bacteria, predominantly Proteobacteria, archaea, fungi, algae, and parasites [93]. In OMZs, the lack of oxygen requires the use of alternative electron acceptors, with nitrate being the most energetically favorable. Nitrate is reduced by denitrifiers to nitrite, which in turn accumulates in OMZs and can fuel anaerobic ammonia oxidation [40]. Presumably then, if functional, viral FocA and NirA would be advantageous during infection by reducing their host’s need to compete for limited nitrate and ammonia.

Finally, two additional features suggested that the viral *nirA* and *focA* are transcribed during host infection. First, both genes were in the same transcriptional unit, similar to what has been reported for *Prochlorococcus* MIT9313, where *nirA*, *focA,* and the uncharacterized gene between them are co-expressed [85]. Second, two NtcA-binding sites followed by sigma-70 promoters were observed upstream of these genes (Supplementary Table S6). In *Prochlorococcus*, these regulatory features were present and more numerous in this same region (triplicate in MIT9313 and duplicate in MIT1323, Supplementary Table S6), which suggests the downstream *nirA* and *focA* genes are likely to be active. Similarly, we posit that the binding sites for NtcA and sigma 70 promote an efficient expression of viral *focA* and *nirA* during infection.

#### Nitrite reductase and nitric oxide reductase

**Fig. 3:**
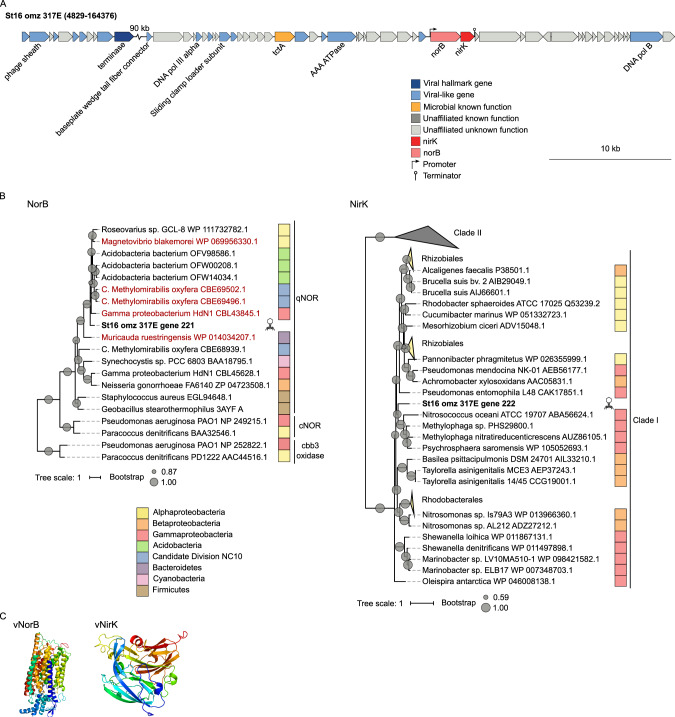
Genomic context, diversity, and protein structure of viral *norB* and *nirK*. **A** Genetic map of the scaffold encoding *norB* and *nirK*. Detailed annotation of this contig can be found in Supplementary Table S2. **B** Maximum-likelihood trees from amino-acid alignments of viral NorB or NirK found in ETSP and reference microbial sequences. The first tree represents the heme–copper oxidases superfamily, including cytochrome C oxidase cbb3-type (cbb3 oxidase), cytochrome c-dependent nitric oxide reductase (cNORs), and quinol-dependent nitric oxide reductases (qNORs) including the potential NO dismutases (in red) (from ref. [101]). Viral AMGs found in this study are highlighted in bold. Internal nodes and SH-like supports are represented by proportional circles (all nodes with support <0.50 were collapsed). **C** Tertiary structures of viral NorB and viral NirK.

The third and fourth of the six N-cycle AMGs included a copper-containing nitrite reductase gene *nirK* and a nitric oxide reductase gene *norB*, neither of which had been previously detected in viral genomes. Similar to the *focA* and *nirA* AMGs, *nirK* and *norB* were contiguous, with *norB* immediately followed by *nirK*, though also with a predicted promoter and terminator (Fig. 3A), which might indicate the kind of transcriptionally autonomous unit (termed a “moron”) that is a hallmark of phage genome evolution [94]. The original viral scaffold containing these new AMGs, St16_omz_317 (52,903 bp), had 68 genes, 22 of them identified as viral-like by VirSorter. Two of these genes corresponded to T4 core genes (gp44 clamp loader subunit and gp43 DNA polymerase), and one to a non-cyanophage T4 core gene (gp9 baseplate wedge tail fiber connector) (Fig. 3A and Supplementary Table S2). Despite the low percent of genes annotated as viral in this scaffold, the high proportion of genes with no affiliation, viral or otherwise, together with the low rate of strand switching (i.e., change of coding strand between two consecutive genes), are also an indicator of a viral sequence, reinforcing the viral origin of this scaffold [52]. The *norB* / *nirK* region likely represents a viral genomic island where host DNA accumulates akin to that in T4-like cyanomyophages [26, 95] as it is surrounded by genes that encode proteins of unknown function, proteins involved in protein biosynthesis and modification, and in tricarboxylate transport (Supplementary Table S2). Taxonomic annotation of the viral-like genes suggested the virus is a Myovirus (Supplementary Table S2), though it is likely a new genus as it formed its own separate viral cluster in gene-sharing networks (Supplementary Table S3) that resolve genus-level taxonomy [54, 96].

The viral NirK protein clustered with microbial NirK from Clade I [97], being most closely related to *Gammaproteobacteria* (67–70% protein identity; Fig. 3B), while NorB was most closely related to *Acidobacteria* (57–62% protein identity), *Magnetovibrio blakemorei* (62% protein identity), and the *Gammaproteobacteria* strain HdN1 (59.3% protein identity) (Fig. 3B). These genes could have been acquired by the virus together or, more likely, in separate events, as the order and orientation of the genes is different from that of any known microbial genome. Interestingly, mobility has been suggested for both of these genes, with *nirK* reported in plasmids from *Azospirillum brasilense* [98], and *norB* present in microbial contigs of four different phylotypes in metagenomes from ETNP OMZ, one of which contained plasmid competence genes upstream of *norB* [99]. The presence of these genes in viral genomes defines an adjunct agent for transferring niche-defining traits in the oxygen-deficient water column.

In silico, both genes appeared to be functional (Supplementary Table S4). Viral NirK contained two copper-binding motifs and key active site residues required for nitrite reducing activity [97] (Supplementary Fig. S6A) and had a strongly supported (100% confidence) structural prediction as a copper-containing nitrite reductase (Fig. 3C and Supplementary Table S4). Viral NorB had similarity (59% protein identity) to a NOD-like NorB from *Gammaproteobacteria* strain HdN1, which is a proposed protein family of length similar to that of the canonical NorB, with 12+ transmembrane domains, but with altered quinol binding and active sites [100, 101]. These sites in the viral “NorB” were also more similar to those from NOD-like NorB enzymes than the canonical NorB (Supplementary Fig. S6B). In contrast, the structural model prediction of the viral “NorB” was of high (100%) confidence to be a nitric oxide reductase (Fig. 3C and Supplementary Table S4), but the absence of a crystal structure of the NOD-like NorB enzyme hindered the discrimination between reductase and dismutase activity using this tool. Interestingly, numerous NOD-like NorB sequences were found in ETNP OMZ waters, with a maximum at the same depth as the second N_2_O peak (140 m) [99]. Since canonical NorB was absent at this same depth, it is suggested that some of these NOD-like NorB enzymes retain their function as nitric oxide reductases [99]. Thus, we interpret this viral NorB to be a NOD-like NorB, with its reductase and/or dismutase activity yet to be determined. Moreover, the pN/pS value for the viral *norB* gene was 0, with 2 SNPs identified, and no SNPs were identified for *nirK* (from one sample with minimal gene coverage >10×) (Supplementary Table S5), suggesting that both genes are likely functional and under strong purifying selection.

Functionally, both AMGs are denitrification genes that could be of benefit to the viruses, with the NirK protein reducing nitrite to nitric oxide, and NorB reducing nitric oxide to nitrous oxide (canonical NorB), or nitric oxide to nitrogen and oxygen (postulated nitric oxide dismutase, NOD-like NorB [101]). Denitrifying bacteria vary in the extent of denitrification genes they encode: some genomes contain all denitrifying genes to be able to reduce nitrate all the way to nitrogen gas, whereas others contain subsets of these genes that enable utilization of specific niches and interactions among partial denitrifiers in a community setting. Thermodynamically, complete and partial denitrification are both theoretically favorable, with Gibbs-free energy (∆G°) of −532 kJ/mol for the complete pathway, −131 kJ/mol for nitrate reduction to nitrite, −105 kJ/mol for nitrite reduction to nitric oxide, −139 kJ/mol for nitric oxide reduction to nitrous oxide, and −314 kJ/mol for nitrous oxide reduction to nitrogen gas (calculated with pyruvate as an electron donor, standardized to the reduction of one mol of the electron acceptor, Supplementary Table S7). From a bioenergetic perspective, though, the proton-motive force generated from complete versus partial denitrification is the same, at most six protons per pair of electrons, since the nitrate reductase is the only denitrification module that translocates protons [102]. However, if denitrifiers were able to make use of nitric oxide dismutation, the amount of energy conserved would improve, translocating 7.3 protons per electron pair, leading to 36.5% energy conservation [102]. Thus, we posit that viral NirK and NorB could benefit the host during infection through the exploitation of specific niche-defining genes or help to safeguard energetically vital pathways in low-redox environments, while the host genome is degraded over the course of infection, thus compensating for the likely elevated energetic cost associated viral production. Additionally, if the viral NorB functions as a nitric oxide dismutase, the increase in energy conservation associated with nitric oxide dismutation would significantly improve the host’s fitness. Future efforts focused on energy budgets and mining SAGs (Single-cell Amplified Genomes) could help resolve these and related hypotheses.

#### Ammonia monooxygenase subunit C

**Fig. 4:**
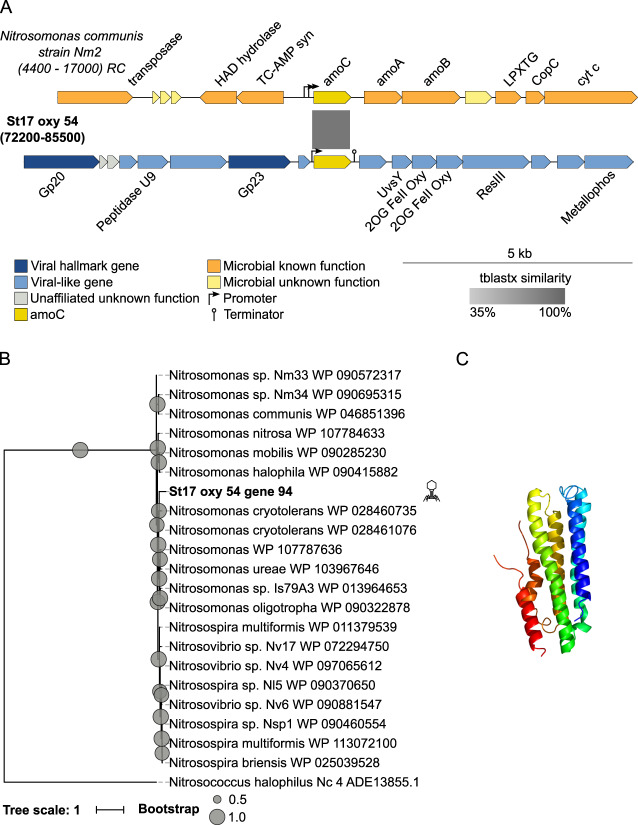
Genomic context, diversity, and protein structure of viral *amoC*. **A** Genetic map of the viral scaffold encoding the bacterial-like *amoC*, and alignment to a reference microbial genome containing this gene. Detailed annotation of the viral scaffold can be found in Supplementary Table S2. **B** A maximum-likelihood tree from an amino-acid alignment of the bacterial-like viral AmoC found in ETSP and reference microbial sequences. The viral AMG found in this study is bolded. Internal nodes and SH-like supports are represented by proportional circles (all nodes with support <0.50 were collapsed). **C** Tertiary structure of the bacterial-like viral AmoC.

The fifth N-related AMG identified was ammonia monooxygenase subunit C gene (*amoC*). In addition to an archaeal version that has been observed in the Tara Oceans Global Ocean Virome (GOV) dataset [30], a recent study described 15 new Thaumarchaeota virus populations that encode viral capsid and thaumarchaeal *amoC* genes. These are potentially tailed viruses that share a common ancestor with related marine Euryarchaeota viruses and are globally distributed in various marine habitats, including OMZs [31]. We found two archaeal-like *amoC* genes and one bacterial-like *amoC* in the ETSP OMZ dataset. These archaeal viruses, represented by scaffolds St14_oxy_254 (35,926 bp) and St17_scm_137 (42,459 bp), corresponded to species “C” and “G” of the previously mentioned 15 new *Thaumarchaeota* virus population [31], and thus will not be described in this section. The bacterial *amoC*-containing viral population, represented by the scaffold St17_oxy_54 (144,618 bp) appeared to be a complete, circularly permuted, T4-like phage genome of 144 kb that encoded 179 genes (Supplementary Table S2). Whole-genome alignments of this scaffold against its closest reference phage, *Pelagibacter* phage HTVC008M, revealed a high degree of homology and synteny between them (Supplementary Fig. S7). It also contained 52 of the 60 hypothesized “core T4” genes [26] (BLASTp *e*-value <0.001). As a reference, 50 hypothesized “core T4” genes were found in *Pelagibacter* phage HTVC008M, with the remaining 10 genes either absent or miss-annotated. The bacterial *amoC* was flanked by a promoter and a terminator and was also located next to the major capsid protein gene Gp23, which is a region known to be an AMG hotspot in T4-like phages (Fig. 4A) [26]. Not surprisingly, this viral contig was clustered with T4-like phages by the gene-sharing network analyses (Supplementary Table S3). Evolutionarily, the bacterial-like *amoC* clusters within the *Nitrosomonas* clade (Fig. 4B), and was most closely related to AmoC from *Nitrosomonas communis*, with 86% amino-acid identity.

Functionally, as posited previously [30], *amoC* could benefit OMZ viruses by enabling access to energy liberated by ammonia oxidation. Across diverse archaea and bacteria, ammonia monooxygenase performs the first step and rate-limiting step in nitrification, aerobically oxidizing ammonia to either nitroxyl or hydroxylamine, respectively [103]. These intermediaries are further oxidized to nitrite, either spontaneously or with the help of additional oxidoreductases. While both ammonia-oxidizing bacteria and archaea are present in the ETSP OMZ [104–106], metatranscriptomic and metaproteomic data suggest that the ammonia-oxidizing archaea, mostly represented by *Nitrosopumilus maritimus*, dominate nitrification in the OMZ [106–108]. The resultant nitrite either continues through nitrification to nitrate in aerobic waters or is converted to nitric oxide or nitrogen gas via denitrification or nitrogen gas via anammox pathways in deeper suboxic waters [33, 109]. While no conserved residues for this enzyme have been described, the high sequence similarity with microbial AmoC suggests that these viral AmoC are likely functional. The pN/pS ratio for the archaeal-like viral *amoC* was 0 for St17_scm_137, with two identified SNPs, and 0.13 for St14_oxy_254, with nine identified SNPs. No SNPs were identified for the bacterial-like *amoC* (from two samples with minimal gene coverage >10×) (Supplementary Table S5), showing a strong purifying selection of the three viral *amoC* genes.

#### GlnK PII regulator

**Fig. 5:**
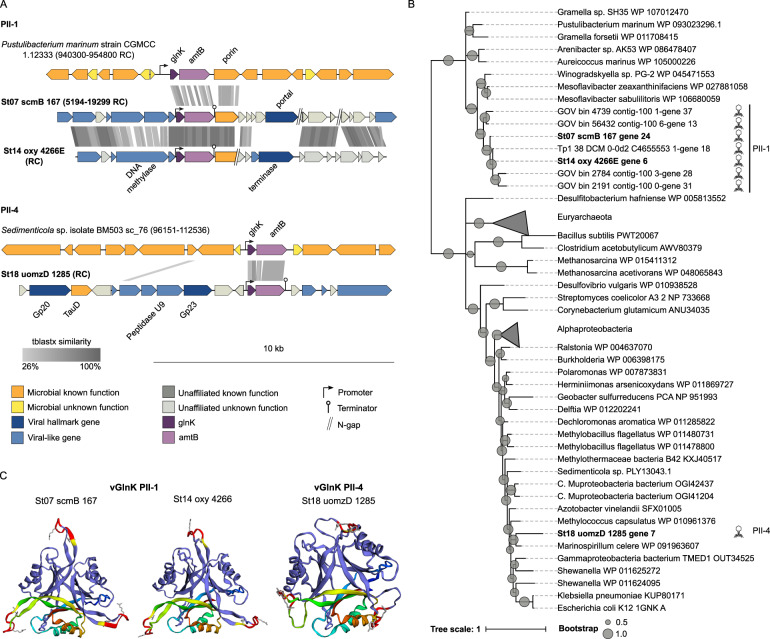
Genomic context, diversity, and protein structure of viral *glnK*. **A** Genetic map of the scaffolds encoding *glnK*, and alignment to reference microbial genomes containing this gene. Detailed annotation of the viral contigs can be found in Supplementary Table S2. **B** A maximum-likelihood tree from an amino-acid alignment of viral GlnK found in ETSP and reference microbial sequences. Viral AMGs found in this study are highlighted in bold. Internal nodes and SH-like supports are represented by proportional circles (all nodes with support <0.50 were collapsed). **C** Tertiary structure of viral GlnK.

The last of the six N-related AMGs was GlnK, a PII signal transduction protein also previously observed in viruses in the GOV dataset [30]. There are three major subgroups of microbial PII proteins, based on gene linkage and similarity at the amino-acid sequence level: *glnB* linked to glutamine synthetase (*glnA*) or NAD synthetase (*nadE*), *glnK* linked to ammonia channel protein (*amtB*), and *nifI* linked to the nitrogenase operon (reviewed in refs. [110] and [111]). Phylogenies from the prior GOV work found viral representatives of the *glnK* and *glnB* subgroups (designated PII-1 and PII-2, respectively), and a functionally ambiguous PII protein that lacked a conserved motif (designated PII-3). PII-1 was widely distributed throughout mesopelagic waters, while PII-2 and PII-3 were geographically restricted to the North Pacific and the South Atlantic Oceans, respectively [30]. We identified three viral populations containing the *glnK* gene followed by the *amtB* gene in the ETSP OMZ dataset (Fig. 5A). The first two belonged to the previously described PII-1 group, and the third was a new viral *glnK* that we designated as PII-4 (Fig. 5B). The first viral scaffold encoding *glnK*, St07_scmB_167 (35,108 bp), contained 66 genes, 26 of them considered viral-like by VirSorter, including one hallmark gene annotated as a portal protein. The second viral scaffold, St14_oxy_4266E (12,943 bp), had 19 genes, with 8 of them corresponding to viral-like genes, including one hallmark gene (terminase). In both cases, there was a predicted promoter upstream of *glnK* and a terminator downstream of the ammonium transporter gene (*amtB*), and a porin gene (*ompL*) immediately downstream of *amtB* (Fig. 5A). These viral contigs clustered together in the gene-sharing network analyses (Supplementary Table S3), but no reference viral genome was assigned to this cluster. The third viral population, represented by St18_uomzD_1285 (15,491 bp), had 18 genes, two of them hallmark viral genes (the major capsid protein Gp23 and the capsid assembly protein Gp20), and 9 viral-like genes. The *glnK* gene was close to the Gp23 gene, similar to the bacterial-like *amoC* viral contig, and a promoter and terminator flanking the *glnK* and *amtB* genes were also predicted. This contig formed a singleton in the gene-sharing network analysis, but the presence of the hallmark structural proteins previously mentioned strongly suggests that it’s a T4-like phage.

Evolutionarily, the *glnK-amtB* genes have been captured by viruses at least two times, one from *Bacteroidetes*, where the new and previously described PII-1 viral *glnK* form a distinct clade, and the second time from *Gammaproteobacteria*, where PII-4 is located (Fig. 5B). The association of *glnK* and *amtB* has been reported as one of the most ancient in biological history [112], with an early lateral transfer event between bacteria and archaea, as proposed by the PII evolution model [111]. Despite the ancient origin of the PII-Amt association, found widespread in Bacteria and Archaea, the PII-Amt-OmpL-like association found in our PII-1 viruses have only been found in *Flavobacteriales* (Fig. 5A). This suggests that the viruses acquired these three genes from a member of *Bacteroidetes*, and it is most likely that their hosts belong to this phylum. The viral porin OmpL had a conserved beta-barrel structure, suggesting that it is functional, though its substrate is unknown. The *glnK* and *amtB* genes from PII-4 were most closely related to *Gammaproteobacteria*, with 68% protein identity, and in this case, no extra genes were incorporated by the phage (Fig. 5A).

In silico, the *glnK* and *amtB* genes from the three viral contigs were identified as PII and Amt, respectively (Supplementary Table S2). The first two PII proteins (PII-1 group) were mostly related to the order *Flavobacteriales* PII, with 71–72% protein identity. The third PII protein (PII-4 group) was mostly related to *Gammaproteobacteria* PII, with 68% protein identity (Fig. 5B). Conserved in the viral GlnK proteins was the uridylylation residue Y51, located in the T-loop, (Fig. 5C and Supplementary Fig. S8), and the tertiary and quaternary structures, predicted with 100 confidence to be PII (Table S4). Unfortunately, pN/pS ratios could not be calculated for the *glnK* genes due to low gene coverage across the samples.

Functionally, OMZ viruses could benefit from the *glnK* and *amtB* genes by regulating the ammonia uptake of their host. AmtB has 11 transmembrane domains and an “N-terminus out” and “C-terminus in” topology [113, 114], and GlnK folds into a four-stranded β-sheet packed against two helices, forming three loops designated the B-, C-, and T-loops [115]. Both form homotrimeric structures, and together they form a complex where the GlnK trimer binds to the cytoplasmic face of AmtB by inserting the T-loop into the cytoplasmic pore exit of an adjacent AmtB subunit, therefore closing the channel [115]. Under nitrogen-limiting conditions, GlnK is covalently modified by uridylylation, inhibiting the interaction with AmtB and therefore enabling the uptake of ammonium. Conversely, under conditions of fixed-nitrogen excess, GlnK is de-uridylylated, enabling its interaction with the ammonium transporter to inhibit ammonium uptake [116, 117]. Ammonium concentration in OMZ decreases with depth (Supplementary Table S1), due to its consumption for cell growth, aerobic oxidation, and anaerobic oxidation. A viral version of the transporter and its regulator could be beneficial, especially in the oxic-anoxic interface, by complementing or replacing the host’s version, thereby increasing its nutrient uptake during infection, as reported in *Ostreococcus tauri* infected by the *O*. *tauri* virus RT-2011 [118]. The viral GlnK might also compete with the host’s PII regulator on its union to the AmtB channel, preventing its closure and therefore ensuring ammonium uptake during infection.

### The biogeochemical and ecological context of virus-encoded N-cycling AMGs

**Fig. 6:**
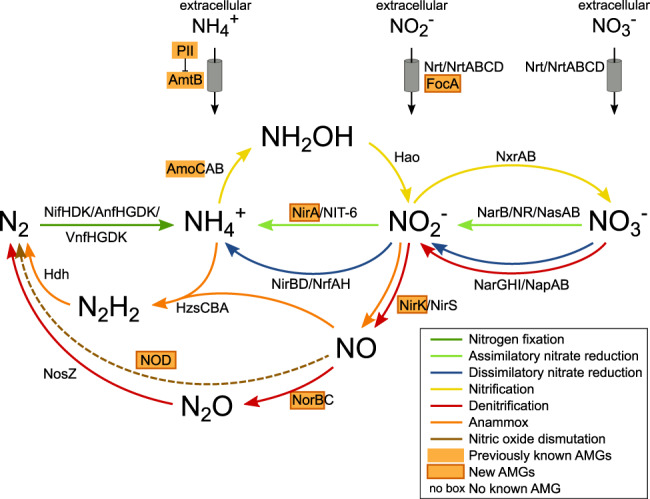
Potential contribution of viruses to nitrogen cycling and transport. The schematic represents the main pathways that drive the nitrogen cycle and the participating enzymes and transporters. Proteins with a viral version are highlighted in orange boxes. Viral NorB and NOD correspond to the same protein that is homologous to both nitric oxide reductase and nitric oxide dismutase. Nitrogen uptake is performed by the ammonia transporter (AmtB, which is regulated by PII), the MFS-type nitrate/nitrite transporter (Nrt), the ABC-type nitrate transporter (NrtABCD), and the formate/nitrite transporter (FocA). The enzymes that transform nitrogen are molybdenum-iron nitrogenase (NifHDK), iron-iron nitrogenase (AnfHGDK), vanadium-iron nitrogenase (VnfHGDK), an ammonia monooxygenase (AmoCAB), hydroxylamine dehydrogenase (Hao), nitrite oxidoreductase (NxrAB), ferredoxin-nitrate reductase (NarB), nitrate reductase (NAD(P)H) (NR), assimilatory nitrate reductase (NasAB), ferredoxin-nitrite reductase (NirA), nitrite reductase (NAD(P)H) (NIT-6), membrane-bound nitrate reductase (NarGHI), periplasmic nitrate reductase (NapAB), nitrite reductase (NADH) (NirBD), cytochrome *c* nitrite reductase (NnfAH), copper-containing nitrite reductase (NirK), heme-containing nitrite reductase (NirS), nitric oxide reductase (NorBC), nitrous oxide reductase (NosZ), hydrazine synthase (HzsCBA), hydrazine dehydrogenase (Hdh), and nitric oxide dismutase (NOD).

The paradigm emerging from studies of viral AMGs is that viruses randomly sample host genetic material, but only a subset of these genes are retained in the viral genomes [119]. In the case of N-cycling AMGs, nitrification (AmoC) and ammonia regulation (GlnK) genes have been previously identified, and to these, we add genes encoding proteins mediating assimilatory nitrite reduction (FocA and NirA) and denitrification (NirK, NorB and/or NOD-like NorB). While these genes were scattered throughout known N-cycling pathways (Fig. 6), we posit that the specific AMGs observed, which are only a subset of known N-cycling genes, are those that represent nutrient or energetic bottlenecks during infection across myriad virus–host pairs in nature. For example, the highly elevated abundances of these genes (particularly *nirK*) in the dysoxic oxycline regions may be consistent with the functioning of these genes in microaerobic environments where some organisms have mechanisms to switch between aerobic respiration and denitrification pathways. The activation of each of these nitrite reduction genes requires elaborate transcriptional regulatory systems [120]. In the case of *nirA*, a nitrite assimilation regulatory gene, it may be that the specific virus–host interaction is augmented by the regulation of the host nitrite reduction pathways, while in the case of *nirK* the host regulatory pathways are most likely implemented for activation of nitrite reduction as no other regulatory regions were apparent in the virus. While many denitrification steps require specific electron carriers, such as ubiquinone, menaquinone, and cytochromes, none were observed in the phages. However, cyanophages that contain photosynthesis AMGs directly linked to electron flow often completely lack any of the related photosynthesis electron carriers [26], so this may simply represent parts of the metabolic machinery that are not prone to turnover and remain intact during viral infection.

**Fig. 7:**
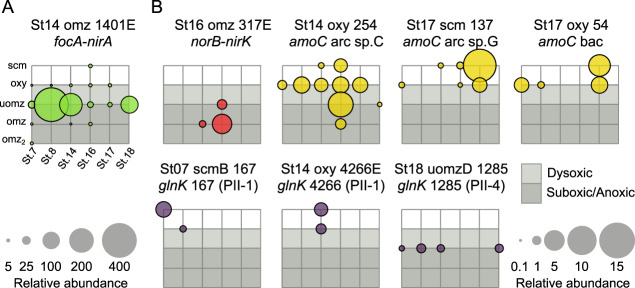
Distribution of N-AMG-containing viral populations across the ETSP OMZ samples. Bubble plots representing the relative abundances, in terms of normalized coverage, of viral populations containing the *focA* and *nirA* genes (panel **A**, in green), *norB* and *nirK* genes (panel **B**, in red), *amoC* genes (panel **B**, in yellow) and the *glnK* genes (panel **B**, in purple). The *x* axis of each grid represents the stations (7, 8, 14, 16, 17, and 18), and the *y* axis represents the sampling depths (from top to bottom: surface chlorophyll maximum (scm), oxycline (oxy), upper OMZ (uomz) and core of the OMZ (omz)). Station 7 had a second core OMZ sample (omz2) and station 18 was only sampled in the upper OMZ. Gray boxes represent the OMZ: light gray for dysoxic waters below the oxycline, and dark gray for suboxic and anoxic waters in the upper and core of the OMZ.

The N-related AMGs were distinctly distributed across the ETSP water column in patterns that mirror those of the key microbial N-metabolisms being modulated (Fig. 7). Some, like the *focA-nirA*-containing viral population, were present at nearly every depth and station, but considerably more abundant in the samples from the upper OMZ that had a deep chlorophyll maximum (normalized coverage of 97.7–382.8, compared to normalized coverage of 1.2–16.7 in the remaining samples with *focA-nirA-*containing viral population) (Fig. 7A). This viral population predicted to be a cyanophage (see above), has abundances notably coincident with those of novel and uncultivated *Prochlorococcus* cyanobacteria that predominate in the secondary chlorophyll maximum of anoxic oceanic OMZs (e.g., Arabian Sea, ETNP, and ETSP) [121–123]. Nitrite reduction is expected to occur in these upper OMZ samples, where nitrite concentration ranges from 0.45 to 11.36 µM (Supplementary Table S1). The other AMG encoding viral populations were less abundant across the dataset and demonstrated strong depth preferences likely corresponding to specific N-related microbes and metabolisms (Fig. 7B). The *norB-nirK*-containing viral population was only found in anoxic waters from stations 14 and 16. As posited earlier, nitrite reduction is expected at these depths, where nitrite concentration varied from 0.45 to 11.36 µM in upper OMZ samples, and from 0.27 to 9.02 µM in OMZ core samples (Table S1), with most of these samples showing nitrite accumulation (>0.5 µM), which occurs only when oxygen falls below 50 nM [124]. Though denitrifiers and anammox bacteria are expected to be found at these depths [33, 40], they were not predicted as hosts for this viral population due to the scant relevant reference genomes for these groups. Similarly, *amoC*-containing viral populations were distributed mostly in surface and oxycline waters, which largely followed the distribution of aerobic ammonia oxidizers [106, 125] (Fig. 7B). Ammonium concentrations at these oxic depths support this first nitrification step, with concentrations that ranged from 75.79 to 779.68 nM in the surface samples, and from 3.48 to 891.01 nM in the oxycline samples. One of the archaeal-like *amoC-*containing viral populations, St14_oxy_254, was also present in OMZ samples from station 16, and the upper OMZ sample from station 18. Even though ammonium concentrations in these samples support ammonium oxidation (from 12.52 to 24.1 nM, Supplementary Table S1), this reaction is not expected in anoxic waters. However, these particular samples were collected at shallower depths (45 and 100 m in station 16, and 66 m in station 18), compared to the rest of the OMZ and upper OMZ samples (Supplementary Table S1), and might be prone to water mixing and intrusion of overlying oxygenated waters. Finally, the *glnK*-containing viral populations (Fig. 7B) were either present in surface and/or oxycline waters from a few stations (PII-1; stations 7 and 8 for St07_scm_167 and station 14 for St14_oxy_4226) or exclusively in upper OMZ waters (PII-4; stations 7, 8, 14 and 18 for St18_uomzD_1285). As posited earlier, ammonium is present in surface and oxycline samples, and in lower concentrations in upper OMZ samples, supporting the presence of PII-1 and PII-4 *glnK*-containing viral populations, respectively (Supplementary Table S1). Considering the wide distribution of the *glnK*-*amtB* genes among Bacteria and Archaea, a particular distribution of these genes across the oxygen gradient is not expected. However, the most closely related microbial *glnK* genes belonged to Proteobacteria (for PII-4) and Bacteroidetes (for PII-1), which represent the first and second most abundant phyla in the OMZs [32].

Stepping back, these findings may have implications for OMZs beyond viruses. First, reduced nitrogen assimilation is known in many cyanobacteria, with nearly all lineages of *Prochlorococcus* encoding *nirA*. The potentially high mobility of genes involved with nitrogen assimilation may be justified by the relatively high concentration and energetic favorability of nitrite (relative to ammonia and nitrate) in this system [126]. Here, we have further identified a cyanophage carrying *focA* and *nirA*, suggesting that N assimilation is also of value to the viruses infecting the novel cyanobacteria from the anoxic secondary chlorophyll maximum [121–123, 126]. Second, this viral manipulation of the denitrification pathway, via NirK, NorB, and/or NOD-like NorB, underlines the relevance of this pathway in anoxic waters. Though the host for the virus carrying these genes could not be predicted, it might be in future studies if parallel microbial metagenomes and single-cell amplified genomes (SAGs) were generated [29]. In this case, we speculate that *Gammaproteobacteria* or *Planctomycetes* might be the hosts, and if so, infection by viruses encoding nitrogen cycling AMGs would promote the denitrification pathway by the reduction of nitrite to nitrous oxide, or the anammox pathway by the reduction of nitrite to nitric oxide, respectively. In any case, infection with the *norB*-*nirK*-containing virus could potentially alter the nitrite, nitric oxide, and nitrous oxide levels within the OMZ, and would have important implications for those trying to assess climate change feedbacks resultant from changes to these ecosystems [127, 128].

In summary, understanding how viruses alter N-related biogeochemical cycling in OMZs is critical, considering the expansion of these suboxic and anoxic water masses and their effects in surface primary production, greenhouse gas emission, and fixed-nitrogen loss [32–34]. Our findings imply that OMZ viruses impact N cycling not only through lysis of key N-cycling microbes but also by modulating diverse N-metabolisms during infection. Such infected “virocells” [10] would be drastically altered in their metabolic capacity and biogeochemical outputs as has been shown now in several environmental model virus–host systems [10, 12, 129]. With these N-related virus AMGs now uncovered, future OMZ virus work can evaluate virocell-impacted nitrogen cycling, as well as develop primer sets for “viral” vs “cellular” versions to differentially quantify the biogeochemical impacts of viruses in OMZ N-cycling genes and transcripts. As standardized practices emerge for viral ecogenomics [130–132], they are enabling the development of global maps of ocean viruses [30, 49, 133] that can be integrated into multi-organism ecological studies [134]. Together these efforts to understand virus-mediated nutrient cycling in climate-critical environments, along with parallel efforts on land (e.g., thawing permafrosts [135, 136]), are now providing quantitative information needed to incorporate viruses into predictive models [137].

## Supplementary information

Supplemental Material document

Table S1

Table S2

Table S3

Table S4

## Data Availability

All high-quality reads and assembled contigs are available on iVirus (CyVerse, 10.25739/mmj5-kt58). Requests for further information should be directed to Matthew B. Sullivan at sullivan.948@osu.edu.
