# Supplementary material for: Potential virus-mediated nitrogen cycling in oxygen-depleted oceanic waters
Source: ISME J. 2020 Nov 16;15(4):981–98. doi: 10.1038/s41396-020-00825-6 (PMC8115048; doi:10.1038/s41396-020-00825-6)
Supplement: Supplementary file 1 — Supplemental Material document [file 41396_2020_825_MOESM1_ESM.pdf]

Potential virus-mediated nitrogen cycling in oxygen-depleted oceanic waters

M. Consuelo Gazitúa[1,2†], Dean R. Vik[1†], Simon Roux[3], Ann C. Gregory[1], Benjamin Bolduc[1], Brittany Widner[4,5], Margaret R. Mulholland[4], Steven J. Hallam[6], Osvaldo Ulloa[7] & Matthew B. Sullivan[1,8*]

[1] Department of Microbiology, The Ohio State University, Columbus, OH 43210, USA
[2] Viromica Consulting, Santiago, Chile
[3] DOE Joint Genome Institute, Berkeley, CA, USA
[4] Department of Ocean, Earth and Atmospheric Sciences, Old Dominion University, Norfolk, VA, USA
[5] Woods Hole Oceanographic Institution, Woods Hole, MA, USA
[6] Department of Microbiology and Immunology, University of British Columbia, Vancouver, BC, Canada
[7] Departamento de Oceanografía & Instituto Milenio de Oceanografía, Universidad de Concepción, Concepción, Chile
[8] Department of Civil, Environmental and Geodetic Engineering, The Ohio State University, Columbus, OH, USA

[†]Authors contributed equally.

*Corresponding author: Matthew B. Sullivan. 484 W 12th Avenue, Riffe Building 947, Columbus, OH, 43210, USA. (614)2471616. sullivan.948@osu.edu.

Supplementary Information

This document includes supplemental figures 1 through 8, supplemental tables 5 to 7, and references. The remaining tables can be found in the following attached spreadsheets: Table_S1.xlsx, Table_S2.xlsx, Table_S3.xlsx and Table_S4.xlsx. Legends for supplementary figures and tables are included individually with each item.

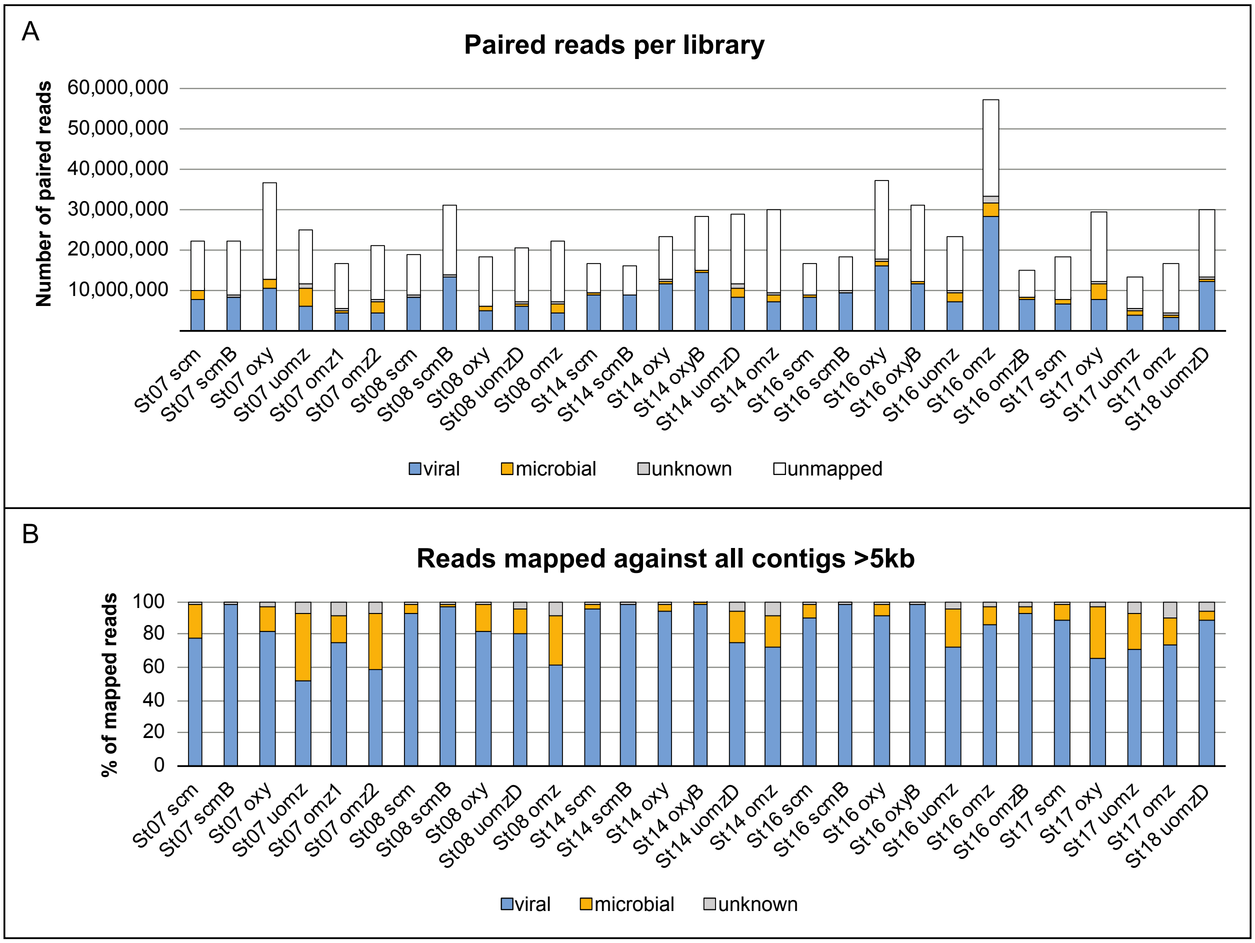

Figure S1: Sequencing depth and read recruitment. A) Plot comparing the sequencing depth, in terms of the number of post-quality control paired end reads, to the number of reads recruited to scaffolds > 5kb identified as viral (blue), microbial (orange) and unknown (gray). B) Percentage of reads recruited to viral, microbial and unknown scaffolds. Samples which names end with “B” represent libraries prepared from viral samples purified with CsCl gradient.

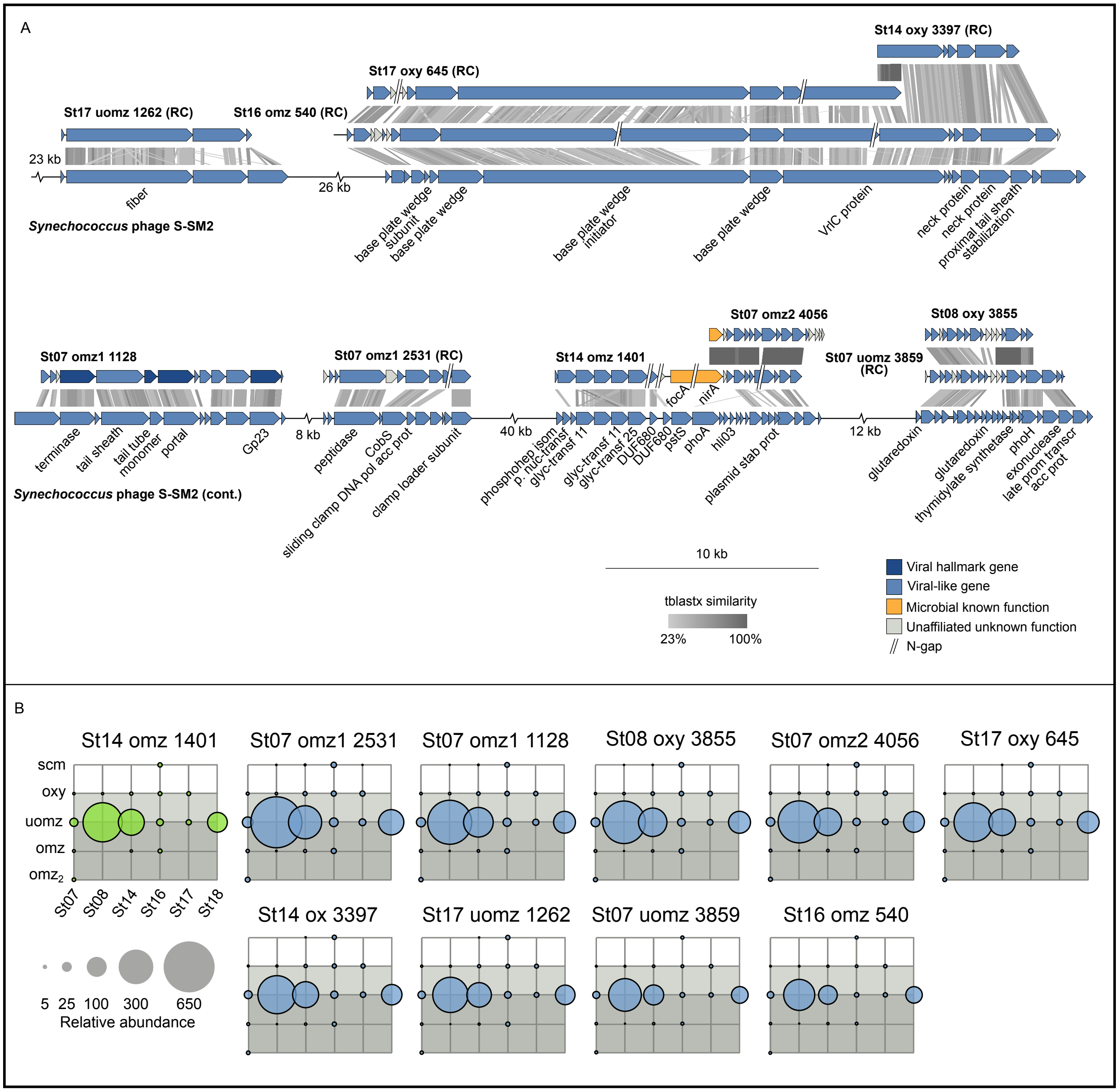

Figure S2: Alignment and distribution of the viral scaffold encoding *nirA* and *focA*, and 9 co-occuring viral scaffolds. A) Genetic map of the *nirA*-*focA*-encoding scaffold and 9 co-occurring viral scaffold, and their alignment to *Synechococcus* phage S-SM2. B) Bubble plots representing the relative abundances, in terms of normalized coverage, of viral populations containing the *focA* and *nirA* genes (in green), and 9 co-occurring viral scaffolds. The x axis of each grid represents the stations (7, 8, 14, 16, 17 and 18), and the y axis represents the sampling depths (from top to bottom: surface chlorophyll maximum (scm), oxycline (oxy), upper OMZ (uomz) and core of the OMZ (omz). Station 7 had a second core OMZ sample (omz2) and station 18 was only sampled in the upper OMZ. Gray boxes represent the OMZ: light gray for dysoxic waters below the oxycline, and dark gray for suboxic and anoxic waters in the upper and core of the OMZ.

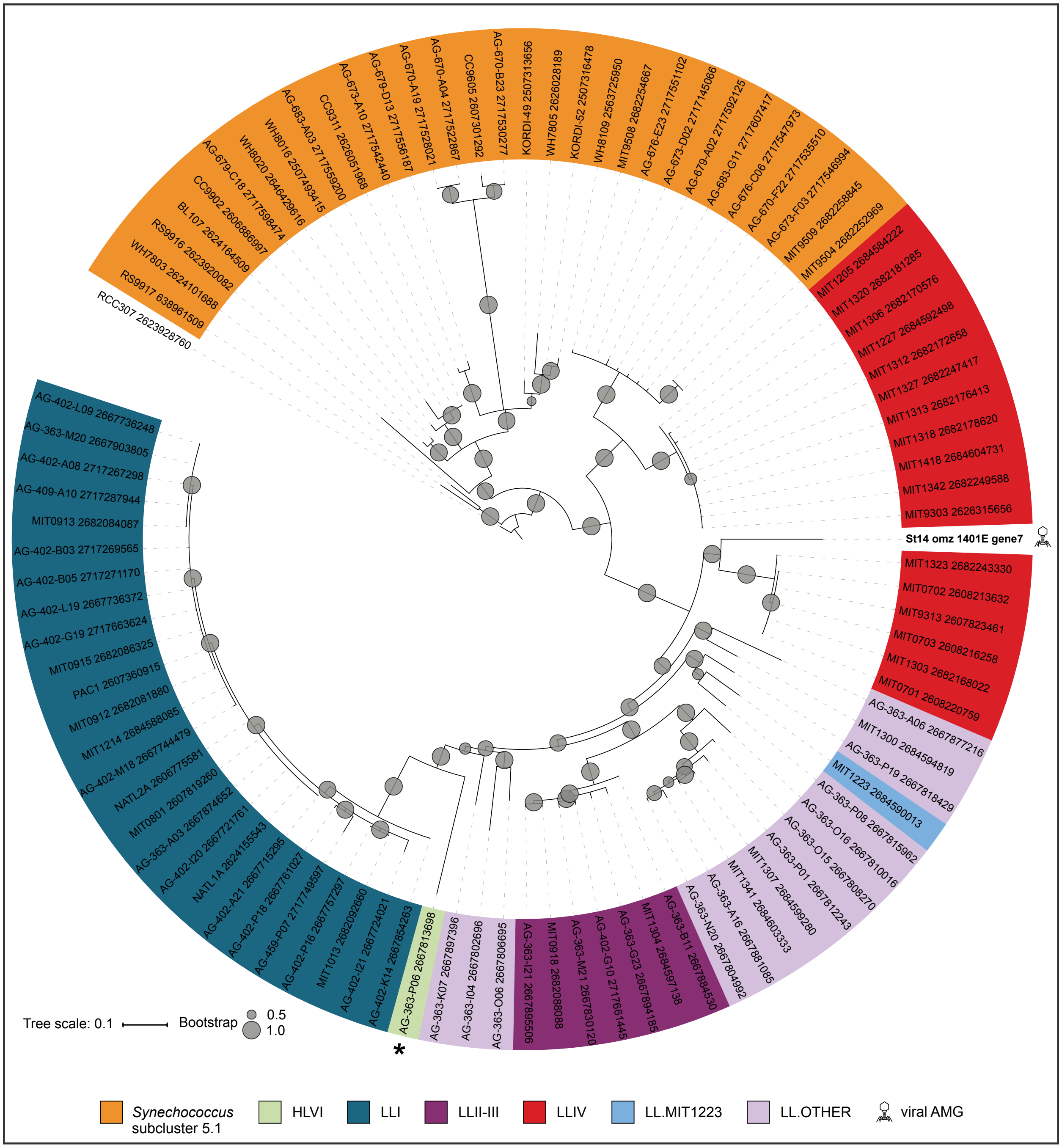

Figure S3: Diversity of FocA. Maximum-likelihood trees from amino-acid alignments of the viral FocA found in ETSP and cyanobacterial sequences. Viral FocA is highlighted in bold. Internal nodes and SH-like supports are represented by proportional circles (all nodes with support < 0.50 were collapsed). Colors represent *Synechococcus* subcluster 5.1, and *Prochlorococcus* high-light (HL) and low-light (LL) adapted clades. The high-light adapted AG-363-P06 single cell, where horizontal gene transfer of *focA* has been proposed, is indicated by an asterisk (1).

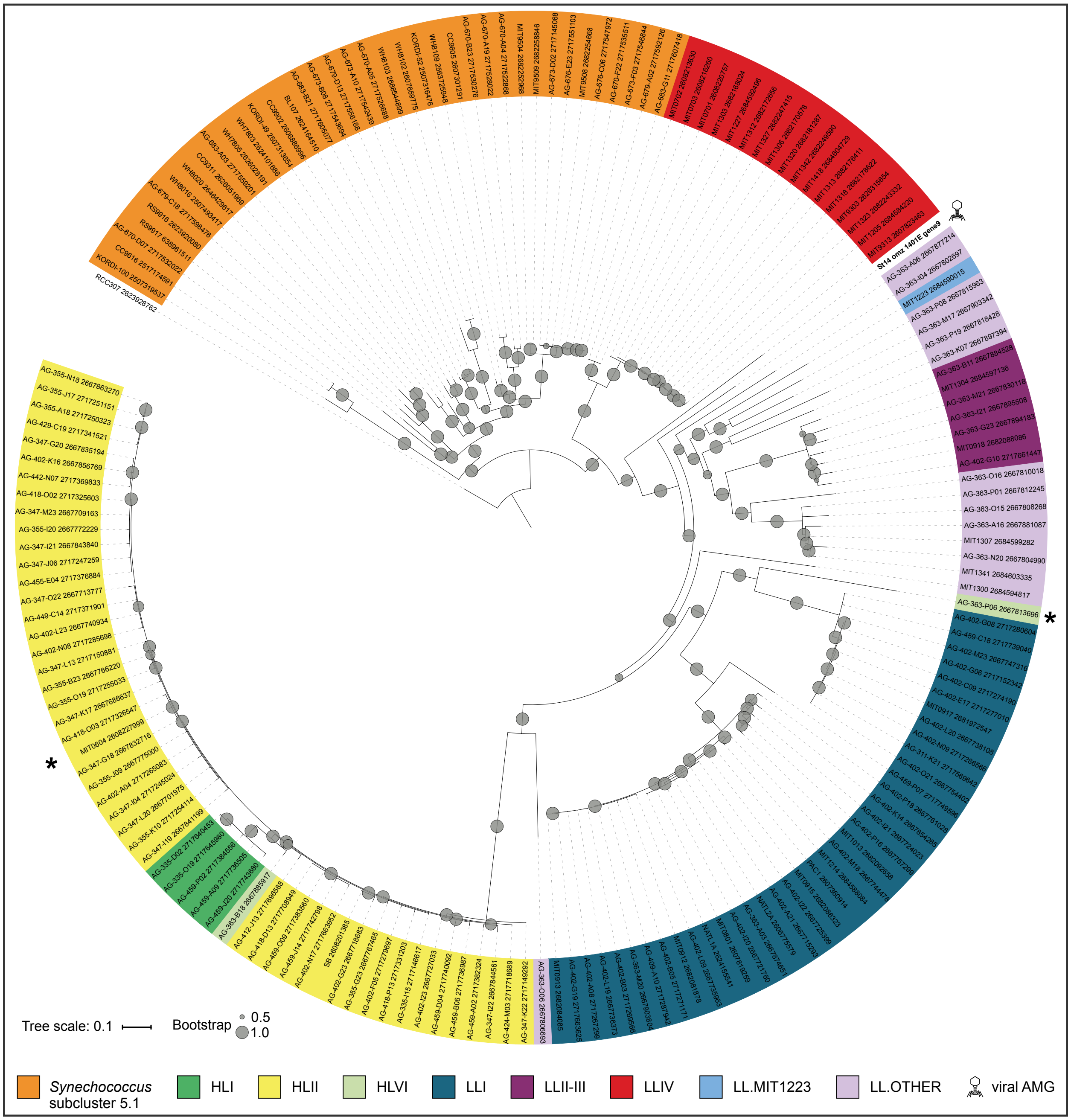

Figure S4: Diversity of NirA. Maximum-likelihood trees from amino-acid alignments of the viral NirA found in ETSP and cyanobacterial sequences. Viral NirA is highlighted in bold. Internal nodes and SH-like supports are represented by proportional circles (all nodes with support < 0.50 were collapsed). Colors represent *Synechococcus* subcluster 5.1, and *Prochlorococcus* high-light (HL) and low-light (LL) adapted clades. Asterisks indicate *Prochlorococcus* sequences where horizontal gene transfer of *focA* has been proposed (1,2).

```
A
WH8109           ----------------MDYVLPNELVDGMIAAGGKKSTVSVKNLLIRGFYSGAILGLAVILALTVGITVKAPFVGSLLFPF
NATL1A           ---------MTSSASQMDYVLPNELVDGMIAAGGKKSSVSVKNLLIRGFYSGAILGLATCLAITIGIQSGMPWLGSFIFPF
MIT0701          ---------MDVNTSQMDYVLPNELVDGMITAGGKKSSVSIKNLLVRGFYSGAILGLATCLAITVGVQSGMPFLGSVLFPF
St14 omz 1401E   MSKVLDGTMKVSASQMDYVLPNELVDGMIAAGGKKATVSIKDLLVRGFYSGAILGLATCLAITVGLQSGLPFLGSVLFPF

WH8109           GFASIVLFGMELVTGNFALLPMATWAGKSTWGATFRNWVWVWIGNWIGTAVVAVIMAISLTS-GTMDGAADNIGPPIWDA
NATL1A           GFASIVLFGMELVTGNFALLPMAVWAGKSSWSATVRNWLWVWIGNFLGTAFVAVLLSISLSSAGNVDPLTAAEGGKGWAV
MIT0701          GFASIVLFGMELVTGNFALLPMATWAGKSTWQATFRNWIWVWIGNFIGCALVALLATSLTSAGTVEPLAAADGGKGWAV
St14 omz 1401E   GFASIVLFGMELVTGNFALLPMATWAGNCSWRQTFRNWTWVWTGNFIGCAVVSILLAISLTSAGTVAPLAAAAGGKGWAA

WH8109           VAQKIMALNQINVEKKYEALGSMGFFLAFLRGLVANWLVCLGVTMALVSKSVPGKILACWLPITAFQSMGMEHIVVNQFL
NATL1A           VAAKIIAIHKANTVVKYEALGSTGFFLAFLRGVIANWLVCLGVTMALVSKSVPGKILACWLPITAFQTMGMEHIVVNMFL
MIT0701          IAAKIMALNKANVVAKYQDLGSTGFFLAFLRGMIANWLVCLGVTMALVSKSVPGKILACWLPITAFQTMGMEHIVVNMFL
St14 omz 1401E   VAAKIIAVNKANVITKYAALGTTGWMLAFLRGMVANWLVCLGVTMALVSKSVPGKILACWLPITAFQAMGMEHIVVNMFL

WH8109           HTAGPILGSGVPFTKVIFWNFLPVTLGNIVGGMVFIGMLFYSTHRTPMDNVLPTEHDEKLERELAAELGAR
NATL1A           HTTGPLLGSGIPFTKVIFWNFLPVTIGNIVGGMVFIGMLFYSTHKTPISNVLPDVKDEKLERELEAQLGAR
MIT0701          HTAGPMLGSGVSFGQVIVWNFIPVTLGNIIGGMVFIGMLFYSTHRTQMSNVLPTVHDEKLERELAAELGAR
St14 omz 1401E   HTTGPLLGSGVGYGSVIFWNFIPVTLGNIVGGMVFIGMLFYSTHRTRMSNVLPTVHDEKLERELAAELGAR

B
Nitrite/sulfite reductase ferredoxin-like half domains
WH8103 (60-130)    MFWRPKTPGKFMLRLRIPNGVLTSNQIRVVASIVERYGENGSCDITTRQNLQLRGILLCDLPEILRRLREA
MIT0604            LFWRPKTPGRFMMRLRVPNGILNSNQLRVIASIVARYGEDGSADITTRQNIQLRGVLINDLPDIIKRLREV
NATL1A             MFWRPKTPGKFMLRLRIPNGIINAEQLKVIASIVARYGENGSCDITTRQNIQLRGVLISDLPEILNRLKKV
MIT0701            MFWRPKTPGKFMLRLRVPNGILSSHQMRVVASIVGRYGEDGSCDITTRQNLQLRGVLLSDLPEILRRLEEA
St14 omz 1401E     MFWRPKTPGKFMLRLRVPNGVLHFNQLEVIADIISKYDG-GRADITTRQNLQLRGILLEDLPEILKSLDSV

WH8103 (312-381)   GINQQKQAGLYFAGIHVPVGRLTAEDLQDIATASLKYGNGEIRLTEDQNIIITGLTSERVEELKTDTLLQ
MIT0604            GINKQKQNNLYFAGLHIPVGRLCVEDIQEIARLSEKYGQSEVRLTEDQNLIIVGLKDNILEEFANEEIIK
NATL1A             GIHSQKQEGKYFAGIHVPVGRLLAEDLQDLANICENFGDKEIRLTEDQNIIITGIDTNLIEEFKEQSILQ
MIT0701            GINPQKQEGLYYAGLHIPVGRLTAEDLQDLASASLHYGNGEIRLTEDQNVILVGLASDNLNSFQADPLLQ
St14 omz 1401E     GIHKQKQKGLYYAGIHIPVGRLDAVDLHRLSLLS-RYGSGEVRLTEDQNVILVNIPEDQLDFVRKDVIIE

Nitrite/sulfite reductase 4Fe-4S binding site
WH8103 (392-456)   AGTVSCTGNTYCSFALTNTKDQSLKAEKELDEELNLPEEIKVHWTGCPNTCGQAYMGAIGLTGTKAK
MIT0604            ASTVSCTGSSYCSFALANTKDIARNISEKLDRELELSEEVKIHWTGCPNNCGQAHMGGIGMTGTKVK
NATL1A             AGTVSCTGNTYCGFALTNTKDQALKISHELDKELDLKDELKIHWTGCPNSCGQAYMGGIGLTGKKAK
MIT0701            AGTVSCTGNTYCSFALTNTKDQAIKMARELDEELELPEELKVHWTGCPNSCGQAYMGAIGLTGTKAR
St14 omz 1401E     AGTVACTGSQFCGFAITDTKGDAERIAKELEYELEIPEEVKIHWTGCPNSCGQAYMGGIGLTGTKVK
                        *      *                                  *    *
```

Figure S5: Protein alignments of FocA and NirA. A) Alignment of FocA from *Synechococcus* WH8109, *Prochlorococcus* NATL1A (LLI), *Prochlorococcus* MIT0701 (LLIV) and the viral scaffold St14 omz 1401E. Transmembrane domains predicted for the viral scaffold (using TMHMM (3)) are underlined and positively and negatively charged amino acid residues from the C-terminal region are indicated by blue and red characters respectively. B) Alignment of the ferredoxin-like domains and the 4Fe-4S binding sites of NirA from *Synechococcus* WH8103, *Prochlorococcus* MIT0604 (HLII), *Prochlorococcus* NATL1A (LLI) *Prochlorococcus* MIT0701 (LLIV) and the viral scaffold St14 omz 1401E. Cysteine residues involved in cofactor binding are indicated by an asterisk.

A

```
R. sphaeroides   PVINEFEMRIIEKEVQLDE-DAYLQAMTFDGSIPGPLMIVHEGDYVELTLINPPENTMPHNIDFHAATGALGGGGLTLIN
A. faecalis      PKVVEFTMVIEEKKIVIDDAGTEVHAMAFNGTVPGPLMVVHQDDYLELTLINPETNTLMHNIDFHAATGALGGGGLTEIN
St16 omz 317E    PKIVEVTMVVDEREIEVAP-GVFTHAFTFNGSVPGPMIVVHEGDYVELTLINSSENMLTHNIDFHASTGAMGGGSLTEVA
G. kaustophilus  PHDVHIEMTAQITDIEIDK-GKIYKAWTFNGQAPGPLVVVNEGDTIHFTLKN-MDPVVPHSMDFHAVHAS-PSKDFIDVM
B. azotoformans  EHEVNVEMTAQITDIEIDK-GKSYKAWTFNGEAPGPLVVVNEGDTINFTLKN-MDPSIPHSMDFHAVHAS-PSKSFIDVM
F. johnsoniae    AVKLKLNMEIKEQEGTMTD-GVKYTYWTFGGSVPGSFIRTRVGDEVEFHLKNHPDNKLPHNIDLHAVTGPGGGATSSLVA
H. denitrificans PAEVDVTLRPEEVTAEVED-GVTFTYTTYNGQVPGPLVRVRQGDTVNLTFENPEENTMPHNVDFHAVAGPGGGAEATMTN
B. pseudomallei  PAKVIVDLEVVEKVLPIAD-GVNYTFWTFGGAVPGNFIRVRQGDTVEFHLKNRPDSKMPHNIDLHAVTGPGGGATSSFTA
Azospirillum sp. PKTHKVALTTTEVESKLDD-GTTYTYWTFNNTVPGPMVRVRVGDTVDVSISNAPESTMNHSVDFHAATGFLGGGQITQVE

R. sphaeroides   PGEKVVLRFKATRAGAFVYHCAPGGPMIPWHVVSGMAGCIMVLPRDGLKDHEGKPVRYDTVYYIGESDHYIPKDEDGTYM
A. faecalis      PGEKTILRFKATKPGVFVYHCAPPG-MVPWHVVSGMNGAIMVLPREGLHDGKGKALTYDKIYYVGEQDFYVPRDENGKYK
St16 omz 317E    PGEQVVLRFKATKSGVFVYHCAPGGAMVPWHVVKGMNGAILVLPREGLTDNKGDAITYDKAYYIGEQDFYLPKDEEGDYK
G. kaustophilus  PNKSGTFTYPANKPGVFMYHC--GTKPVLQHIANGMHGVIIVKPKNGYPTDK----EVDREYVLIQNEWY-------KYN
B. azotoformans  PNESGTFTYKASNPGVFMYHC--GTKPVLAHIANGMHGVIIVKPKNGYPTDN----QIDKEFVIVQNEWY-------KYN
F. johnsoniae    PGHEKVFSFKVINPGLYVYHCAT--APVGMHIANGMYGLILVEPEGGLP-------PVDKEYYVMQGDFYTQGEYGEK--
H. denitrificans PGETAHLRFKATYPGAYIYHCAV--PNMDMHISAGMFGLILVEPPEGLP-------EVDHEIYLGQHELYTDKDAGEE--
B. pseudomallei  PGHESRFTFKALNEGLFVYHCAT--APVGMHVANGMYGLILVEPPEGLP-------KVDREYYVMQGDFYT----NGKYR
Azospirillum sp. PGQTKAFSFKALTPGVYVYHCAT--PMVAHHISKGMYGLIVVEPEAGLA-------PVDHEFYVMQQEIYATKAKNLA--
                               *   *               *  *                               *
R. sphaeroides   RFSDPSEGYED-MVAVMDTLIPSHIVFNGAVGALTGEG---ALKAKVGDNV--LFVHSQPNRDSRPHLIGGHGDLVWETG
A. faecalis      KYEAPGDAYED-TVKVMRTLTPTHVVFNGAVGALTGDK---AMTAAVGEKV--LIVHSQANRDTRPHLIGGHGDYVWATG
St16 omz 317E    RYDFAIEGMND-ELKLMKQLTPSHIVFNGAVGALTGDN---ALTANVGETV--LFLHSQANRATYPHLIGGHGDYVWERG
G. kaustophilus  DMNDFQNGVPSYVVFSSKALKPGDPNTNGDTFTLKEK----PLLAKVGEKIRLYINNVGPNEVSSFHVVGTVFDDVYLDG
B. azotoformans  DLDDMTNGVPSQVVFSTKALKEGQPNTNGDTYSLKEK----PLLAKVGDRIRIYVNNVGPNEVSSFHVVGTVFDDVYIDG
F. johnsoniae    -------GLQPFDMNKAVKETPDYVVFNGKVGSLTNGG---ELTAKVGETVRLFVGNGGPNLVSSFHVIGEIFDSVHIEG
H. denitrificans -------GQHTFDYEAMRNEDPTYVLMNGEKYAWTPNGRGPAATVGTGETVRVYFVDGGPNLSSSFHPIGSVWETLYPEG
B. pseudomallei  E-----KGLQSFDMDKAIDERPTYVVFNGAEGALTGER---AMRARTDETVRLFVGNGGPNLVSSFHVIGAVFDKVRADG
Azospirillum sp. -------NAED-DYDGLVNERPSYLVFNGTVGALTKDK---PLKAKVGETVRLFFGVGGPNLTSSFHVIGEIFDRVYNLG

R. sphaeroides   KFHNAPERDLETWFIRGGSAGAALYKFLQPGVYAYVNHNLIEAVHKGATAHVLVE
A. faecalis      KFNTPPDVDQETWFIPGGAAGAAFYTFQQPGIYAYVNHNLIEAFELGAAAHFKVT
St16 omz 317E    SFDNAPQVNLESWVIGAGSAGAAVYTFKQPGLYAYVNHNLIEAILLGAAAHVQVK
G. kaustophilus  NPNNHLQ-GMQTVMLPASGGAVVEFTVTRPGTYPIVTHQFNHA-QKGAVAMLKVT
B. azotoformans  NPANHYK-GMQTVMLPASGGAVVEFTVVEEGSYSIVTHQFNH-VQKGAAAIIKVT
F. johnsoniae    G--STINKNVQTTLIPAGGAAIVDFKVETPGTFILVDHSIFRAFNKGALGMLKVE
H. denitrificans SLTTEPQTHIQTRQVPPGSTTIATMSSPVPGDFKLVDHSLSRVVRKGCMAVVRAE
B. pseudomallei  S--NVTQNDVQTTLIPAGGAATIEFHTRVPGNYTFVDHSIFRAFNKGALAILKVD
Azospirillum sp. GLESEPAKGIQTVTVAPGGATMVEFKVDYPGKYALVDHALSRA-TKGLIGILEVE
                                               *
```

B

	Quinol binding site		Catalytic site			
	321	745	507	558	580	
G. stearothermophilus	MFGALLAHYYTEPDSFFG	PDT	IHLWVEG	GHHY	LEV	qNOR
N. gonorrhoeae	LLGGLTAHYTVEGQGFYG	PDL	VHLWVEG	LHHL	LEV	
M oxyfera CBE68939	IVGAAVAHYRAEPGKFYG	GDA	VHLWVEG	GHHW	MEV	
Synechocystis sp.	LMGMVTAHYAVEGEGFYG	GDL	VHLWVEG	LHHL	LEV	
HdN1 NorZ1	FIGGFTAHYTVEGQTFYG	GDV	VHLWVEG	FHHL	LEV	
S. aureus	LLGELLAHYYVE-NKFFG	WDI	VHLWVEG	GHHY	LEV	
M. oxyfera CBE69496	LAGILSAEDFVGGGPGSA	GGA	IHMWVEV	SHNF	MQV	NOD
M. oxyfera CBE69502	LAGILGAEDFVGGGPGEA	GGV	IHMWVEV	SHNF	MQV	
M. ruestringensis	SSGFVTINEFVDYLGF--	GAC	VHMWVEA	SHNF	LQF	
HdN1 NorZ2	FAGIAAAWDFVKP-----	GIA	VHMWVEV	SHNF	LQV	
St16 omz 317E	VAGILGAMDFANP-----	GGL	VHMWVEV	AHNF	LQI	

Figure S6: Protein alignment of NirK and NorB. A) Trimmed multiple sequence alignment of NirK from *Rhodobacter sphaeroides* ATCC 17025, *Alcaligenes faecalis* S-6, *Geobacillus kaustophilus* HTA426, *Bacillus azotoformans* LMG 9581 T, *Jonesia denitrificans* DSM 20603, *Propionibacter acnes* C1, *Flavobacteria johnsoniae* UW101, *Halomonas denitrificans* ATCC 35960, *Burkholderia pseudomallei* 668, *Azospirillum* sp. B506 and the viral NirK. Sequences belonging to Clade I NirK are in bold. Copper binding motifs T1Cu and T2Cu are indicated in green and blue characters respectively, active site residues Asp and His required for nitrite reducing activity in yellow characters and conserved regions are indicated by an asterisk (from (4)). B) Amino acid sequence comparison of the quinol-binding and catalytic sites in quinol-dependent nitric oxide reductases (qNOR) and putative nitric oxide dismutases (NOD), including the viral NorB. Numbering above the alignment refers to the first amino acid and corresponds to the residue numbers of *Geobacillus stearothermophilus*. Strongly conserved residues are indicated in red characters, and specific changes in these residues are indicated in green characters (from (5)).

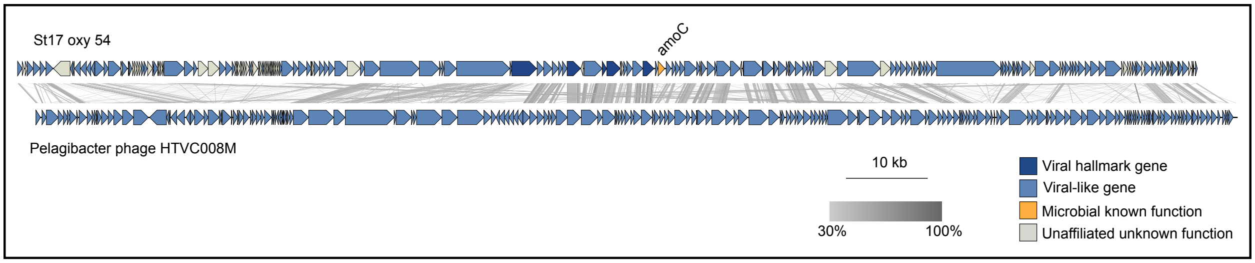

Figure S7: Alignment of the bacterial-like *amoC* viral scaffold to a reference viral genome. Genetic map of the viral scaffold St17_oxy_54, aligned to *Pelagibacter* phage HTVC008M genome.

E. coli K12	--MKLVTVIIKPFKLEDVREALSSIGIQGLTVTEVKGFGRQKGHAELYRGAEYSVNFLPKVKIDVAIADDQLDEVIDIVSKAAYTGKIGDGKIFVAELQRVIRIRTGEADEAAL----
M. celere	--MKLVTAIIKPFKLDDVREALSDMGVQGITVTEVKGFGRQKGHTELYRGAEYVVDFLPKVKLEIAVDDDQLEGVIDAIVKVANTGKIGDGKIFVSPLDQVIRIRTGETGKDAV----
Gammaproteobacteria TMED1	--MKLVTAIIKPFKLDDVRDALSDISIQGMTVTEVKGFGRQKGHTELYRGAEYVVDFLPKVKVEVAIEDDNLDSVIEAITNASNTGKVGDGKIFVTPLDNVIRIRTGETGGEAV----
Methylothermaceae B42	--MKLVTAVIKPFKLDDVREALSDVGVAGITVTEVKGFGRQKGHTELYRGAEYVVDFLPKVKIEVAVSDEQLDQVVEAVLNSARTGKIGDGKIFVSSLEQVIRIRTGESGAEAL----
C. Mupro. RBG 16 64 11	--MKLVTAIIKPFKLDDVRDALAEVGVQGITVTEVKGFGRQKGHTELYRGAEYVVDFLPKVKLEIAIDAKQLDKVVEAIVKAARTDKIGDGKIFVYDLEQVVRIRTGETGKDAL----
C. Mupro. RBG 16 62 13	--MKLVTAIIKPFKLDDVRDALAEIGVQGMTVTEVKGFGRQKGHTELYRGAEYVVDFLPKVKIEIAIDAKQLDRVIEAVTKSARTDKIGDGKIFVLDLEQVVRIRTGESGKGAL----
**St18 uomzD 1285**	**--MKLVTAVIKPYKLDDVRDVLADMEVSGLTVTEVKGYGRQKGHTELYRGAEYTIDFLPKIKIELLIDADHLDAVLNEIIKTTKSGKFGDGKIWVSDLENVIRIRTGESGKEAI----**
M. sabulilitoris	--MKKVEAIIRKSKFSAVKKALHEVGVNFFSYWDVTGLGNEK-IGHVYRGVSYSTSDIQRRYLSIVVNDEFLDVTVQAIIEAGKTGDVGDGKIFVSNIEEVYRIRTGEKGGNTLN---
Arenibacter sp. AK53	--MKKIEAIIRKSRFDDVKKALHQIEVNFFSYWDVTGVGNEK-QGHVYRGISYSTTDIQRRYLSIVVSDEFLERTIHTILETAYTGNVGDGKVFVSEIQEAYRIRTKERGHAGIN---
M. zeaxanthinifaciens	--MKKVEAIIRKSKFSAVKKALHEVGVNFFSYWDVTGLGNEK-IGHVYRGVSYSTSDIQRRYLSIVVNDEFLDVTVQTIIEAGKTGEVGDGKIFVSNIEEVYRIRTEEKGGNTLN---
Gramella sp. SH35	--MKKIEAIIRKSKFDEVKKALHDTEVNFFSYWDVTGVGNEK-QGHVYRGVSYSTSDIQRRYLSIVVSDEFVEKTVKVILETAKTGTVGDGKIFVTPVDEAYRIRTGEKGTDSLN---
Winogradskyella sp. PG-2	--MKKVEAIIRKSKFSKVKNALHEVGVNFFSYWDVTGLGNEK-EGHVYRGVSYSTSDIQRRYLSIVVNDDFEDVTVKAILEAGATGDVGDGKIFVSHIEEVYRIRTGEKGGQTLK---
A. marinus	--MKKVEAIIRKSKFDDVKKALHNIEVNFFSYWDVTGVGNET-QGHVYRGITYSTSDIQRRYLSIVVSDEFLDRTVEAILDSAVTGNVGDGKIFVSEIQEAYRIRTRENGKAGIN---
**St07 scmB 167**	**--MKKVDAIIRKSQFDDVKSALLAVKVTFFSYWDCTGVGNEK-LGISYRGVEYSTSDIQRRYLSIVVSDEFLDRTIKAILEAGKTGNIGDGKIFVSNIEEAYRIRTGERGNQTLN---**
**St14 oxy 4266E**	**--MKKVDAIIRKSQFDDVKSALLAVKVTFFSYWDCTGVGNEK-LDRRYRGIEYSTSDIQRRYLSIVVSDEFLDRTIKAILESGKTGNIGDGKIFVSNIEEAYRIRTGERGNQTLN---**

Figure S8: Protein alignment of GlnK. Alignments of a reference cyrstalized GlnK sequence (IGNK from *Escherichia coli K12*), the viral GlnK sequences and their most closely related homologs from *Marinospirillum celere*, *Gammaproteobacteria TMED1*, *Methylothermaceae B42*, *C. Muproteobacteria RBG 16 64 11, C. Muproteobacteria RBG 16 62 13*, *Mesoflavibacter sabulilitoris*, *Arenibacter sp. AK53*, *Mesoflavibacter zeaxanthinifaciens*, *Gramella sp. SH35*, *Winogradskyella sp. PG-2* and *Aureicoccus marinus*. The T loop residues E44–Y51 are highlighted in red, with the tip of the loop highlighted in yellow. Viral sequences are bolded.

Table S5: N-AMGs pN/pS values. Ratio of non-synonymous to synonymous polymorphisms (pN/pS) of the AMGs across the ETSP OMZ samples.

AMG (gene)	Sample	gene coverage			gene length	pi	refSNPct[1]	obsSNP[2]	pN/pS	average pN/pS
		average	min.	max.						
focA (St14 omz 1401E gene 7)	St07_omz1	18	11	32	936	0.0018	7	7	0	0
	St07_omz2	41	25	59	936	0.0019	7	7	0	
	St07_oxy	37	22	53	936	0.0018	7	7	0	
	St07_uomz	267	157	319	936	0.0017	7	7	0	
	St08_uomzD	4653	3086	5007	936	0.0017	7	7	0	
	St14_omz	23	10	36	936	0.0017	7	7	0	
	St14_oxy	33	18	49	936	0.0017	7	7	0	
	St14_uomzD	2962	2120	3260	936	0.0018	7	7	0	
	St16_omz	133	114	151	936	0.0019	7	7	0	
	St16_oxy	71	49	89	936	0.0018	7	7	0	
	St16_scm	45	23	57	936	0.0018	7	7	0	
	St16_uomz	166	116	191	936	0.0018	7	7	0	
	St17_oxy	60	41	76	936	0.0017	7	7	0	
	St17_uomz	50	33	63	936	0.0018	7	7	0	
	St18_uomzD	1492	1168	1632	936	0.0017	7	7	0	
nirA (St14 omz 1401E gene 9)	St07_omz2	47	24	70	1509	0.0044	27	27	0.2146	0.2482
	St07_uomz	232	149	306	1509	0.0046	27	27	0.2194	
	St08_uomzD	4670	2695	6385	1509	0.0047	27	27	0.2464	
	St14_omz	31	16	48	1509	0.0045	27	27	0.2556	
	St14_oxy	37	14	64	1509	0.0046	27	27	0.2520	
	St14_uomzD	2913	1822	3857	1509	0.0046	27	27	0.2498	
	St16_omz	122	72	158	1509	0.0044	27	27	0.2847	
	St16_oxy	94	37	157	1509	0.0047	27	27	0.2384	
	St16_scm	47	20	73	1509	0.0045	27	27	0.2845	
	St16_uomz	161	97	216	1509	0.0046	27	27	0.2693	
	St17_oxy	75	30	116	1509	0.0045	27	27	0.2334	
	St17_uomz	54	20	80	1509	0.0047	27	27	0.2295	
	St18_uomzD	1620	951	2126	1509	0.0046	27	27	0.2498	
norB (St16 omz 317E gene 221)	St16_omz	75	55	102	2604	0.0001	2	2	0	0
nirK (St16 omz 317E gene 222)	St16_omz	75	60	93	1125	n.a.	0	0	n.a.	n.a.
*amoC*_bac (St17 oxy 54 gene 94)	St17_oxy	25	18	32	813	n.a.	0	0	n.a.	n.a.
	St17_scm	23	17	36	813	n.a.	0	0	n.a.	n.a.
*amoC*_arc_sp.C (St14 oxy 254 gene 41)	St08_oxy	18	11	23	564	0.0015	9	9	0.0528	0.1158
	St14_oxy	14	10	18	564	0.0008	9	9	0.0727	
	St16_omz	34	24	46	564	0.0036	9	9	0.1649	
	St16_oxy	40	26	53	564	0.0038	9	9	0.1377	
	St16_uomz	67	54	77	564	0.0042	9	9	0.1453	
	St17_oxy	25	18	37	564	0.0027	9	9	0.1211	
*amoC*_arc_sp.G (St17 scm 137 gene 29)	St17_oxy	16	13	20	564	0.0009	2	2	0	0
	St17_scm	57	45	71	564	0.0007	2	2	0	

(1) refSNPct: number of SNPs identified in the gene.
(2) obsSNP: number of SNPs with coverage.
(3) n.a.: uncalculated pN/pS ratio (observed ratio of nonsynonymous to synonymous mutations / expected ratio of non-synonymous to synonymous mutations) due to an expected ratio of 0.

Table S6: *nirA* operon promoter region. NtcA binding site and sigma 70 promoter of the *nirA* operon found in cyanobacteria and in the *focA*-*nirA*-containing ETSP virus. NtcA binding sites and sigma 70 promoters are highlighted in bold, and sigma 70-like promoters are underlined.

Genome	NtcA binding site and 31 bp downstream	Distance of NtcA site from *nirA* start codon (bp)
PCC7942	**GTAGTTTCTGTTAC**CAATTGCGAATCGAGAACTGCC**TAATCT**GC	65
WH8103	**GTAATTCCATCAAC**AGAACAACTTTTGAGTACGAAC**TAGAAA**AGG	346
WH8102	**GTAATTCCATCAAC**AGAACAACTTTTGAGTACGAAC**TAGAAA**AGG	347
MIT9313	**GTTCAATCTGATAC**CGCCAATACCCTCTTCCATCAGGCT**TAATGA**	468, 311, 154
MIT1323	**GTTCAATCTGATAC**CGCCAATACCCTCTTCCATCAGGCT**TAATGA**	312, 155
St14 omz 1401E	**GTATCAACACTTAC**AAAAGTGTATCATAGTGA**TACACT**TTTTTCT	191
St14 omz 1401E	**GTAATCTGCTTTAC**TACTTTGGAACTACTAG**TACTAT**TTGCTGCT	136

Table S7: Standard Gibbs free energy (ΔG°) calculated for denitrification and its individual pathways. Values were calculated for 25°C with pyruvate as an electron donor (reduced to 1 or 2 e^- transferred, equivalent to the reduction of one mol of the electron acceptor) and unit activity for all reactants and products using Thermodyn© (6).

Reaction	Equation	ΔG° (kJ/mol)
Denitrification	$NO_3^- + {}^1/_2CH_3COCOO^- \rightarrow {}^1/_2N_2 + {}^3/_2HCO_3^-$	-532
Nitrate reduction	$NO_3^- + {}^1/_5CH_3COCOO^- + {}^1/_5H_2O \rightarrow NO_2^- + {}^3/_5HCO_3^- + {}^2/_5H^+$	-131
Nitrite reduction	$NO_2^- + {}^1/_{10}CH_3COCOO^- + {}^4/_5H^+ \rightarrow NO + {}^3/_{10}HCO_3^- + {}^2/_5H_2O$	-105
Nitric oxide reduction	$NO + {}^1/_{10}CH_3COCOO^- + {}^1/_{10}H_2O \rightarrow {}^1/_2N_2O + {}^3/_{10}HCO_3^- + {}^1/_5H^+$	-139
Nitrous oxide reduction	$N_2O + {}^1/_5CH_3COCOO^- + {}^1/_5H_2O \rightarrow N_2 + {}^3/_5HCO_3^- + {}^2/_5H^+$	-314

Supplementary References

1. Berube PM, Rasmussen A, Braakman R, Stepanauskas R, Chisholm SW. Emergence of trait variability through the lens of nitrogen assimilation in Prochlorococcus. *Elife* 2019; **8**: e41043.
2. Berube PM, Biller SJ, Kent AG, Berta-Thompson JW, Roggensack SE, Roache-Johnson KH, et al. Physiology and evolution of nitrate acquisition in Prochlorococcus. *ISME J* 2015; **9**: 1195–1207.
3. Krogh A, Larsson B, Von Heijne G, Sonnhammer ELL. Predicting transmembrane protein topology with a hidden Markov model: Application to complete genomes. *J Mol Biol* 2001; **305**: 567–580.
4. Helen D, Kim H, Tytgat B, Anne W. Highly diverse nirK genes comprise two major clades that harbour ammonium-producing denitrifiers. *BMC Genomics* 2016; **17**: 155.
5. Ettwig KF, Speth DR, Reimann J, Wu ML, Jetten MSM, Keltjens JT. Bacterial oxygen production in the dark. *Front Microbiol* 2012; **3**: 273.
6. Damgaard LR, Hanselmann K. Thermodyn - A spread sheet for the calculation of free reaction energies under actual conditions. Based on Hanselmann KW 1991. Microbial energetics applied to waste repositories. Experientia 1991; **47**: 645-687. Birkhäuser Verlag, CH-4010 Basel/Switzerland.
